# Identification of SUMO conjugation sites in the budding yeast proteome

**DOI:** 10.15698/mic2017.10.593

**Published:** 2017-10-02

**Authors:** Miguel Esteras, I-Chun Liu, Ambrosius P. Snijders, Adam Jarmuz, Luis Aragon

**Affiliations:** 1 Cell Cycle Group, MRC London Institute of Medical Sciences, Du Cane Road, London W12 0NN, UK.; 2 Protein Analysis and Proteomics Platform, The Francis Crick Institute, 1 Midland Road, London NW1 1AT, UK.

**Keywords:** SUMO, proteome, budding yeast, mass spectrometry, site-specific SUMOylation

## Abstract

Post-translational modification by the small ubiquitin-like modifier (SUMO) is an important mechanism regulating protein function. Identification of SUMO conjugation sites on substrates is a challenging task. Here we employed a proteomic method to map SUMO acceptor lysines in budding yeast proteins. We report the identification of 257 lysine residues where SUMO is potentially attached. Amongst the hits, we identified already known SUMO substrates and sites, confirming the success of the approach. In addition, we tested several of the novel substrates using SUMO immunoprecipitation analysis and confirmed that the SUMO acceptor lysines identified in these proteins are indeed bona fide SUMOylation sites. We believe that the collection of SUMO sites presented here is an important resource for future functional studies of SUMOylation in yeast.

## INTRODUCTION

SUMO (small ubiquitin-related modifier) is a 10-kDa highly conserved protein modifier that reversibly conjugates to specific lysine residues on many target proteins 1. The functional consequences of SUMO modification include changes in protein stability, localization, DNA-binding, or protein interactions. These SUMO effects can be mediated by providing a new binding interface, masking existing binding sites, or inducing conformational changes on the substrates 2. SUMO is covalently conjugated to its substrates sequentially by the action of an E1 activating enzyme, an E2 conjugating enzyme, and an E3 ligase 1. Similar to ubiquitin, SUMO itself can be further SUMOylated via addition of SUMO moieties to lysine residues on SUMO 3. SUMO can also be released from substrates by SUMO proteases (Ulp1 and Ulp2 in yeast).

A single SUMO gene is found in *S. cerevisiae* (*SMT3*), *C. elegans* (*SMO-1*) and *D. melanogaster* (*smt3*). In contrast, the human genome contains five SUMO variants, *SUMO1-SUMO5*. *SUMO2* and *SUMO3* are 97% similar each other but only share 50% of sequence similarity with *SUMO1*. Accordingly, *SUMO1* and *SUMO2/3* seem to be functionally distinct with different substrate set 4,5,6 role has been yet identified for SUMO4 and even its aptness to be conjugated *in vivo* remains unclear 7.

A consensus motif for SUMOylation was proposed soon after the mapping of the first SUMO-modified lysine residues. Studies of the first conjugation sites suggested that the acceptor lysines were contained within the consensus ψKxE (where ψ is a large hydrophobic amino acid and x any amino acid) 8. This motif together with a particular 3D structure in the substrate was proposed to allow the binding of the E2 enzyme, Ubc9, and the consequent transfer of SUMO 9. In addition to the simple 4 amino acid consensus motif, two more extended versions have also been identified. The first one was the phosphorylation-dependent SUMOylation motif (PDSM), which consists of the core motif succeeded by a phosphorylated serine and a proline (ψKxExxpSP) 10. The second extended motif is the negatively charged amino-acid dependent SUMOylation motif (NDSM), consisting of the core motif succeeded by two or more acidic amino acids in the C-terminal tail 11. Although previously described motifs are found in many substrates, some exceptions have been identified, e.g. the K14 in E2-25K (*H. sapiens*) and the K164 in PCNA (*S. cerevisiae*). It is still unclear how Ubc9 recognizes these sites. Whether these unorthodox motifs mimic the 3D structure present at the SUMO consensus motif, or whether they are no more than rare exceptions remains to be answered. However, they make predictions of whether and where SUMOylation might occur in a given protein challenging.

The identification and quantification of SUMOylation by mass spectrometry (MS) is specially challenging. Most of the *in vivo* modified proteins have low steady-state SUMOylation and conjugated SUMO is very likely to be lost during the protein extraction and purification. Hence, input protein sample for MS are likely to contain low amounts of SUMOylated peptides. In addition, SUMO-modified lysines keep an amino acid (aa) side chain (5 residues in case of Smt3) after trypsin digestion which belongs to the SUMO modifier. During tandem MS, this aa side chain generates overlapping fragment ions with the ones from the target protein peptide. Standard database matching logarithms find it challenging to assign correct sequences to such a complex ion spectrum. Therefore, our knowledge of site-specific SUMOylation of proteomes is poor particularly when compared to other PTMs, like phosphorylation.

Several studies in mammalian systems have used clever strategies to improve the identification of SUMOylation sites. One of these strategies involves the mutation of all internal lysines in SUMO to arginines to make the mutant SUMO immune to digestion when Lys-C protease is used 12. This allows digestion of the entire lysate and enrichment of SUMOylated peptides, greatly diminishing the sample complexity. In this study, we have used a similar proteomic approach to identify SUMO-modified proteins and their conjugation sites in the budding yeast proteome. We report over 200 potential SUMO sites. We have chosen a handful of newly identified SUMOylated substrates and demonstrate that mutation of the identified SUMO-conjugation sites prevents their modification *in vivo*. We present this resource to aid future efforts in the functional characterisation of SUMOylation in these yeast proteins.

## RESULTS

### Strategy to enrich SUMO-bearing peptides

**Figure 1:**
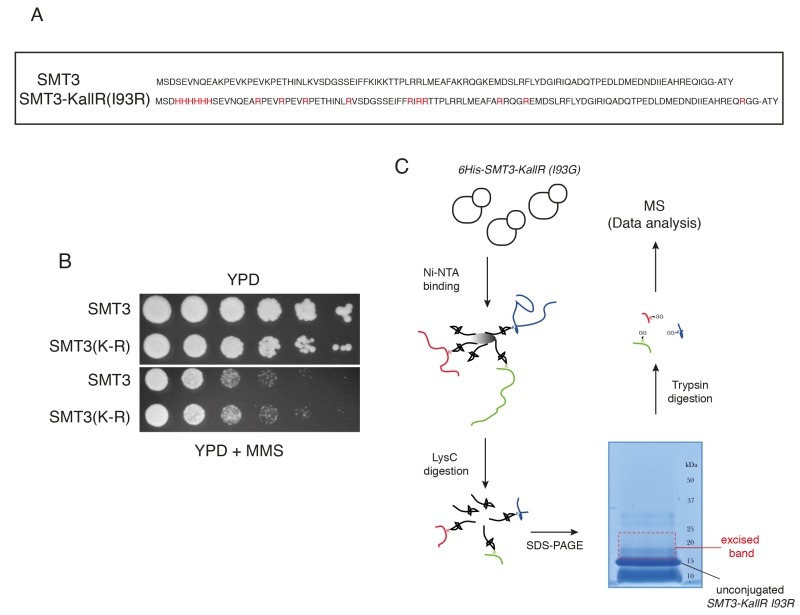
FIGURE 1: Proteomic screen to identify SUMO sites in budding yeast proteins. **(A)** Sequences of wildtype SUMO (*SMT3*) and the lysine-deficient His6 tagged mutant (*SMT3-KallR I93R*) used in the method. **(B)** Wild-type and *SMT3-KallR I93R* strains plated on full media (YPD) and media containing methyl methanesulphonate (MMS). **(C)** Diagrammatic representation of the purification strategy employed to enrich for *SMT3*-conjugated peptides. Cell lysates from yeast expressing *SMT3-KallR I93R* were digested with the endoprotease LysC, cleaving after lysines. The digested lysates were run on SDS-PAGE. Unconjugated *SMT3-KallR I93R* (indicated) and *SMT3-KallR I93R* conjugated to target protein fragments were identified. Gel area above the unconjugated *SMT3-KallR I93R* band were excised, digested with trypsin, and analyzed by nano LC-MS/MS. And database searched to identify SUMO acceptor lysines in the yeast proteome.

To identify SUMOylation sites in the yeast proteome we adapted a strategy recently used to discover novel acceptor lysines for SUMO2 in Hela cells 12. We used a His6-tagged *SMT3* (yeast SUMO) allele where all lysines had been replaced by arginines, *SMT3-KallR* (Fig. 1A). Similar alleles have been employed previously to identify sites of poly-SUMO chain formation 3. In addition a mutation at the C-terminus of Smt3 was introduced, isoleucine at position 96 was substituted by arginine (*SMT3-KallR-I96R*; Fig. 1A) 13. Importantly, we confirmed that the SMT3-*KallR-I96R* allele is conjugated *in vivo* (Fig. S1) and able to support growth similar to its wild-type Smt3 counterpart (Fig. 1B). Smt3-KallR-I96R protein is unsensitive to digestion by endoprotease LysC, an enzyme that specifically cleaves after lysine residues. Therefore unconjugated Smt3-KallR and Smt3-KallR covalently attached to peptides from target proteins can be easily separated by SDS-PAGE from the rest of the proteome fragments after digestion of protein extracts with LysC (Fig. 1C). Excision of the gel area above unconjugated Smt3-KallR selectively isolates Smt3-modified peptides (Fig. 1C). The excised gel fragments containing the peptides modified were digested with trypsin, which cleaves after arginine and lysine, and therefore removes most of Smt3-KallR-I96R from the substrate pep-tides. This strategy generates diglycine-modified isopeptides that are more compatible with mass spectrometry identification compared to wildtype conjugates.

### Identification of SUMO-acceptor lysines by mass spectrometry 

**Figure 2:**
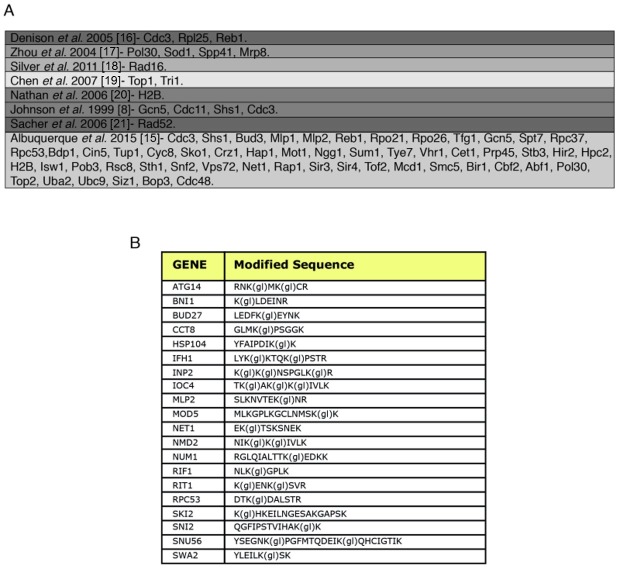
FIGURE 2: SUMO-acceptor lysines detected in our mass spectrometry analysis were compared with SUMO-acceptor lysines already described in previous studies done in *S. cerevisiae*. **(A)** Previous studies were based on either MS or site-directed mutagenesis/immunoblotting, or a combination of both. Most of the SUMO-acceptor lysines previously found were also detected in this study. SUMO substrates (total of 257) identified in the yeast proteome of cells grown asynchronously. Previously published SUMO-substrates were obtained 8,15,16,17,18,19,20,21. **(B)** To ensure that diglycine-modified lysines detected by mass spectrometry after Smt3 purification are not due to modification by ubiquitin or Nedd8, a centromeric plasmid 8His-SMT3-KallR-REQIGG-pRS415 expressing the Smt3 variant used previously for the Smt3 purification protocol with the difference that the RGG conjugating terminus was replace for the native RIEQGG C-terminus was employed. SUMO-acceptor lysines modified by the 8His-Smt3-KallR-REQIGG keep a side chain of 5 aa after trypsin digestion (EQIGG). Therefore, any diglycine-modified lysines detected by mass spectrometry under these conditions can only be due to either false positive hits, or to ubiquitinated or neddylated contaminants. A large culture of 9 l of the strain expressing the 8His-SMT3-KallR-REQIGG variant was grown in YPD and harvested at O.D. 0.9. Smt3 purification and mass spectrometry analysis was performed as described in Material and Methods. We detected 23 diglycine-modified lysines. None of these corresponded with previously detected diglycine-modified lysines in our Smt3-RGG pulldowns. In addition none of these diglycine-modified lysines are within SUMO consensus sequences. This strongly indicates that sumo-acceptor lysines identified after purification of Smt3-RGG pulldowns represent bona fide SUMOylation sites.

Smt3 purifications for mass spectrometry analysis were performed using 9 liters of cells (harvested at O.D. 0.9) grown in YPD. The Smt3 purifications were performed as described in Material and Methods. Lys-C digested peptides were separated by size in a SDS-PAGE gel. The area that corresponds to peptides between 15 and 25 kDa (containing peptides conjugated to Smt3) was cut from the gel (Fig. 1C), fragmented in smaller horizontal bands and digested with trypsin. These fractions were digested and loaded on the mass spectrometer separately, so that the complexity of the peptide mixture could be reduced. Trypsin digested peptides were analyzed by nanoscale liquid chromatography coupled to high-resolution mass spectrometers (LTQ-Orbitrap Velos) and identified using MaxQuant/Andromeda software 14. A total of 257 SUMO-acceptor lysines were identified on asynchronous growing yeast cultures (Table 1 & Suppl. Table S2). We were able to detect many SUMO-acceptor lysines previously described in several studies for yeast proteins (Fig. 2A). Interestingly, our SUMO dataset had a significant overlap (over 50 proteins/sites) with that reported in a recent study were a different strategy was followed to identify site-specific SUMOylation 15. Ubiquitination or neddylation also produce diglycine-modified lysines after trypsin digestion, therefore to ensure that our strategy was able to enrich for diglycine-modified lysines that represented SUMO conjugation of Smt3-KallR-I96R we used an Smt3-KallR where I96 was not mutated to arginine. We identified just over 20 diglycine-modified lysines in this sample (Fig. 2B). None of these diglycine-modified lysines were placed within a SUMO consensus sequence. This result suggests that the large majority of diglycine-modified lysines in the strains carrying Smt3-KallR-I96R (Table 1) are indeed the product of SUMO-conjugation.

**Table 1:** Summary of all sumo-acceptor lysines detected by mass spectrometry.

**Gene**	**Modified Sequence**	**Gene**	**Modified Sequence**
ABD1	NISPIIK(gl)IR	RPC37	SIDNK(gl)LFVTEEDEEDRTQDR
ABF1	MDK(gl)IVVNYYEYK	RPC53	EPTPSVK(gl)TEPVGTGLQSYLEER
ABF1	QQGVTIK(gl)NDTEDDSINK	RPC53	GFIK(gl)SEGSGSSIVQK
ABF1	VSNDSK(gl)LDFVTDDLEYHLANTHPDDTNDK	RPC53	LPAFERPAVKEEK(gl)EDMETQASDPSK
ADE1	SITK(gl)TELDGILPLVAR	RPC53	MAK(gl)YLNNTHVISSGPLAAGNFVSEK
AIM44	LNMEK(gl)DIK	RPC53	PAVK(gl)EEKEDMETQASDPSK
ALY2	FAPLDK(gl)VTLHR	RPC53	VK(gl)LEEESK
AOS1	MDMK(gl)VEKISEDEIAIYDRQIR	RPC82	LK(gl)TEDGFVIPALPAAVSK
AOS1	SIIEVTTRKDEEDEK(gl)K	RPD3	DAEDLGDVEEDSAEAK(gl)DTK
AOS1	VEK(gl)LSEDEIALYDR	RPL13A/B	GFTLAEVK(gl)AAGLTAAYAR
APA1	ALTFFQDWLNENPELK(gl)K	RPL18A	APK(gl)GQNTLILR
APC5	K(gl)K(gl)TDELLESLSVEEDR	RPL18A/B	ALK(gl)QEGAANK
ARP7	LAPLIK(gl)EENDMENMADEQK	RPL18A/B	VVLK(gl)ALFLSK
ARP8	LTK(gl)EIKDLEGHYVNAPDK	RPL20A	DIK(gl)FPIPHR
ASG1	LLSNIK(gl)TER	RPL20A	TADVK(gl)R
ATG2	DEPVSQK(gl)ISK	RPL25	APK(gl)YASK
BAF1	QQGVTIK(gl)NDTEDDSINK	RPL25	AVK(gl)ELYEVDVLK
BDP1	ARQEFK(gl)PLHSLTKEEQEEEEEK	RPL25	LDSYK(gl)VIEQPITSETAMK
BDP1	DK(gl)LLNADIPESDRK	RPL28	FVSK(gl)LAEEK
BDP1	K(gl)AHTAIQLK	RPL28	IPNVPVIVK(gl)AR
BDP1	K(gl)TEVVLGTIDDLK	RPL34A/B	AFLIEEQK(gl)IVK
BDP1	KGSGGIMTNDLK(gl)VYR	RPL35B	EQIASQIVDIK(gl)K
BDP1	NTAK(gl)EEDQTAQR	RPL4A/B	LNPYAK(gl)VFAAEK
BFR2	SIADQISDIAIK(gl)PVNK	RPL5	VAAK(gl)IAALAGQQ
BIR1	EISGIK(gl)KETDDGK	RPL7A	VTK(gl)ATLELLK
BIR1	ILEDVSVK(gl)NETPNNEMLLFETGTPIASQENK	RPL8A/B	NFGIGQAVQPK(gl)R
BIR1	VIK(gl)PEFEPVPSVAR	RPL8B	LVSTIDANFADK(gl)YDEVKK
BMH2	IVSSIEQK(gl)EESKEK	RPO21	VDLLNTDHTLDPSLLESGSEILGDLK(gl)LQVLLDEEYK
BNI1	K(gl)LDEINR	RPP1B	ALEGK(gl)DLK
BOP3	IGASAVAALNDNISIK(gl)EEDVAR	RPP2A	VSSVLSALEGK(gl)SVDELITEGNEK
BRE1	KIK(gl)LELSDPSEPLTQSDVIAFQK	RPS0A/B	TWEK(gl)LVLAAR
BRX1	AEAAVERK(gl)IK	RPS1	VSGFK(gl)DEVLETV
BSP1	NIK(gl)KEEEDSIPEAIK	RPS10A	HEEIDTK(gl)NLYVIK
BUD27	LEDFK(gl)EYNK	RPS13	K(gl)GLTPSQIGVLLR
BUD3	FFEIEEELK(gl)EELK	RPS17A/B	GISFK(gl)LQEEER
BUD3	NK(gl)QENINSSSNLFPEGK	RPS17A/B	YYPK(gl)ITIDFQTNK
BUD3	TGNEDVGNNNPSNSIPK(gl)IEKPPAFK	RPS1A	VTGFK(gl)DEVLETV
BUD4	AGNK(gl)QENNEINIKAEEEIEPMTQQETDGLK	RPS20	RYIDLEAPVQIVK(gl)R
BUD4	QENNEINIK(gl)AEEEIEPMTQQETDGIK	RPS20	SDFQK(gl)EKVEEQEQQQQQIIK
CBF2	DNQPIK(gl)KEENIVNEDGPNTSR	RPS20	SDFQKEK(gl)VEEQEQQQQQIIK
CBF5	EDFVIK(gl)PEAAGASTDTSEWPLLLK	RPS21A	ADDHASVQINVAK(gl)VDEEGR
CBF5	VNENTPEQWK(gl)K	RPS24A/B	TQFGGGK(gl)SVGFGIVYNSVAEAK
CDC11	EAKIK(gl)QEE	RPS28A/B	MDSK(gl)TPVTIAK
CDC12	IRLNGDLEEIQGK(gl)VK	RPS3	ALPDAVTIIEPK(gl)EEEPILAPSVK
CDC12	K(gl)YFTDQVK	RPS31	LIFAGK(gl)QLEDGR
CDC12	YK(gl)EEENALK	RPS31	TLSDYNIQK(gl)ESTLHLVLR
CDC19	IIVK(gl)IENQQGVNNFDEILK	RPS6A	K(gl)GEQELEGLTDTTVPK
CDC3	FEAAESDVK(gl)VEPGLGMGITSSQSEK	RPS8A/B	NVK(gl)EEETVAK
CDC3	KLQK(gl)SETELFAR	RPS9B	K(gl)AEASGEAAEEAEDEE
CDC3	SLK(gl)EEQVSIK	RPT6	K(gl)IEFPPPSVAAR
CDC3	SLKEEQVSIK(gl)QDPEQEER	RPT6	YGEPQK(gl)VVLK
CDC48	EVK(gl)VEGEDVEMTDEGAK	RRG9	RIIK(gl)SNWKR
CET1	KIAGNAVGSVVK(gl)KEEEANAAVDNIFEEK	RRP15	IFNAIIATQVK(gl)TEK
CIN5	MTDTAFVPSPPVGFIK(gl)EENK	RRP9	TIDEYNNFDAGDLDK(gl)DIIASR
CMD1	SSNITEEQIAEFK(gl)EAFAIFDK	RSC2	TSVK(gl)RESEPGTDTNNDEDYEATDMDIDNPK
CMR1	IFIFTDDSGTIK(gl)QEE	RSC4	LIAKPETVQSEVK(gl)NER
CMR1	LSDLIK(gl)DEDESALLEK	RSC58	VK(gl)QEELLNTNEEGINR
CRZ1	IESGIVNIK(gl)NELDDTSK	RSC8	LENNGNSVK(gl)K
CRZ1	PK(gl)IESGIVNIK	RSC8	PFLPENVIK(gl)QEVEGGDGAEPQVKK
CWC15	NK(gl)VEDK	RTF1	NAEHVK(gl)KEDSNNFDSK
CYC8	QPTHAIPTQAPATGITNAEPQVK(gl)K	RVB1	K(gl)EIVVNDVNEAK
DAD3	MEHNISPIQQEVIDK(gl)YK	SAT4	DLK(gl)PENLLLTHDGVLK
DEP1	LSSLVK(gl)QETLTESLK	SCC2	K(gl)SEIVSRPEAK
DUN1	IVFGK(gl)SCSFIFK	SCM4	TLK(gl)PESER
EAF7	EVK(gl)FEDEEK	SDC1	SVTNQNVK(gl)IEESSSTNSVIEESSEPK
EBP2	SQELK(gl)KEEPTIVTASNLK	SEF1	DSK(gl)VSVQTYLSR
ENO2	IEEELGDK(gl)AVYAGENFHHGDKL	SGS1	QLENDIK(gl)LEVIR
ERG10	AGAK(gl)FGQTVLVDGVER	SHS1	EIK(gl)QENENLIR
ESC1	VNEGEEPEHQAVDIPVKVEVK(gl)EEQEEMPSK	SHS1	FLNSPDLPERTK(gl)LR
FBA1	DYIMSPVGNPEGPEK(gl)PNK	SHS1	SIK(gl)TESSPK
FHL1	HPQNTTTDIENEVENPVTDDNGNLK(gl)LELPDNLDNADFSK	SIC1	LTDEEK(gl)R
FLP1	EMIALK(gl)DETNPIEEWQHIEQLK	SIR2	IK(gl)VAQPDSLR
GCN2	LMIDSPHLK(gl)K	SIR3	K(gl)IK(gl)IEPSADDDVNNGNIPSQR
GCN4	FIK(gl)TEEDPIIK	SIR4	APFIK(gl)SESKPFSSDALSK
GCN4	TEEDPIIK(gl)QDTPSNLDFDFALPQTATAPDAK	SIZ1	NFLQNALVVGK(gl)SDPYR
GCN5	VK(gl)LENNVEEIQPEQAETNKQEGTDK	SIZ1	STNTDILTEK(gl)GSSAPSR
GPD1	PFK(gl)VTVIGSGNWGTTIAK	SIZ1	TLDPK(gl)SYNIVASETTTPVTNR
GPM1	GLVK(gl)HLEGISDADIAK	SIZ1	VIPEYLGNSSSYIGK(gl)QLPNILGK
GSH1	ASGEIPTTAK(gl)FFR	SKO1	DTNVVK(gl)SENAGYPSVNSRPIILDK
GZF3	AISNVK(gl)TETTPPHFIPFLQSSK	SLI15	EVK(gl)NYYQSPVR
HAA1	IGSQENSVK(gl)QENYSK	SLI15	NNVYMNTLK(gl)YEDK
HAP1	VK(gl)QESSDELKK	SMC5	LDDIVSK(gl)ISAR
HDA1	MDSVMVK(gl)K	SNF2	DIGAELK(gl)R
HHF1	K(gl)ILRDNIQGITKPAIR	SOD1	GDAGVSGVVK(gl)FEQASESEPTTVSYEIAGNSPNAER
HHT1	RFQK(gl)STELLIR	SOD1	K(gl)THGAPTDEVR
HHT1	STGGK(gl)APRK	SPA2	TIK(gl)REEEDEDFDRVNHNIQITGAYTK
HHT1	YK(gl)PGTVAIR	SPC24	LLK(gl)DLDGLER
HMO1	DAIIAAPVK(gl)AVR	SPP41	GVTTPIK(gl)IEDSDANVPPVSIAVSTIEPSQDK
HMO1	TTDPSVK(gl)IK	SPP41	IPEIK(gl)NESVDLGSNITDILSSTITNILPEITATDVK
HMS1	DSSLLSAASIVK(gl)KEQLSGFENFLPLSK	SPP41	PK(gl)SEDHEWPLSDSSASQNYDAHLK
HPC2	MQTQTDTNAEVLNTDNSIK(gl)K	SPP41	RPQIK(gl)PEVSVINLVQNLVNTK
HSC82	KPK(gl)IEEVDEEEEEK	SPP41	VK(gl)QQLDK
HTA1/2	ATK(gl)ASQEL	SPT15	DGTKPATTFQSEEDIK(gl)R
HTB1/2	AVTK(gl)YSSSTQA	SPT7	NGFGTVIK(gl)QEDDDQIQFHNDHSINGNEAFEK
HTB1/2	KPASK(gl)APAEK	SSE1	GK(gl)LEEEYAPFASDAEK
HTB2	SSAAEK(gl)KPASK	SSM4	LSPK(gl)DLK
IES4	EPADEDPEVK(gl)QLEK	STB3	EVSPPQAISVK(gl)SEASSSIFSK
IES4	GSEFTASDVK(gl)GSDDK	STH1	LIQLDELPK(gl)VFR
IES4	K(gl)KEPADEDPEVK	STH1	VFREDIEEHFK(gl)KEDSEPIGR
IES4	SQESSVLSESQEQLANNPK(gl)IEDTSPPSANSR	STP1	IK(gl)SEVNAK
IKI1	DIK(gl)DENR	SUM1	IITIK(gl)SSSENSGNNTTNNNNTDNVIK
INO80	SIAVIINKEDK(gl)DISDFSK	SUM1	IK(gl)NEIPINSLLPSSK
IRC20	K(gl)LEEADDK	SUM1	K(gl)TPGDEETTTFVPLENSQPSDTIRK
ISW1	AK(gl)IEDTSNVGTEQLVAEK	SUM1	LPSGPK(gl)DDVDTLALTSAQNQANSLR
ISW1	DIISPLLLNPTK(gl)R	SUM1	VNVEENK(gl)TEK
ISW1	LK(gl)EEGSR	SUM1	YFVEPSTK(gl)QESLLLSAPSSSR
KAP123	TSLLQTAFSEPK(gl)ENVR	SWA2	YLEILK(gl)SK
KRR1	DFIAPEEEAYK(gl)PNQN	SWC3	TTAESTQVDVK(gl)K
LIF1	ISNQSVIK(gl)MEDDDFDDFQFFGISK	SWI3	IQKEEEPENNTVIEGVK(gl)EESQPDENTK
MAG1	IK(gl)REYDEIIK	SWR1	AGGEQDLADLK(gl)FR
MCD1	ELSEEK(gl)EVIFTDVLK	SWR1	LLAQAEDEDDVK(gl)AANLAMR
MCM1	QQPQQQQPQQQQQVINAHANSIGHINQDQVPAGAIK(gl)QEVK	SWR1	YDHIAK(gl)VEEPSEAFTIK
MET12	MEMLRNTGLEK(gl)	TAF12	SAIFK(gl)QTEPAIPISENISTK
MET28	VAATTAVVVK(gl)EEEAPVSTSNEIDK	TAF14	TGSASTVK(gl)GSVDLEK
MET4	MK(gl)QEQSHEGDSYSTEFINIFGK	TAH11	EK(gl)MPDSQANLMDRLR
MGA2	ALK(gl)EEEEDEHENK	TDH1/2/3	TASGNIIPSSTGAAK(gl)AVGK
MLP1	KIK(gl)TEDEEEKETDK	TDH2/3	VVDLVEHVAK(gl)A
MLP2	RVK(gl)EEYDIWQSR	TEC1	K(gl)IENFIK
MPP10	VK(gl)LDLFADEEDEPNAEGVGEASDK	TEF1	LPLQDVYK(gl)IGGIGTVPVGR
MRP8	EFK(gl)DIPDLK	TFG1	AVDSSNNASNTVPSPIK(gl)QEEGLNSTVAER
MRPL11	IK(gl)QTGGKLTK	TFG1	ENESPVK(gl)KEEDSDTLSK
MRPL22	SSMK(gl)KATLLLR	TFG1	GSLVK(gl)KDDPEYAEEREK
MZM1	K(gl)VDGSSTKEPR	TFG1	VK(gl)DEDPNEYNEFPLR
NCB2	LHHNSVSDPVK(gl)SEDSS	TFP1	AIK(gl)EESQSIYIPR
NET1	DIDNSK(gl)PDPR	TIF4631	SAEPEVK(gl)QETPAEEGEQGEK
NET1	EKEDTNDK(gl)LLEK	TOA1	IEVK(gl)PEIELTINNANITTVENIDDESEK
NET1	IK(gl)SSIVEEDIVSR	TOF2	FKPTGETK(gl)VQK
NET1	ISEIEK(gl)ELKEGPSSPASILPAK	TOF2	LHQSQGK(gl)EALFR
NET1	K(gl)IKSSIVEEDIVSR	TOF2	LVEKEFPDK(gl)SLGAASSTSHAK
NET1	K(gl)SQAEPSGIVEPK	TOP1	IK(gl)TEPVQSSSLPSPPAK
NET1	K(gl)VRPSLSSLSDLVSR	TOP1	KIK(gl)KEDGDVK
NET1	NEIDLDDSAPVSLYK(gl)SVK	TOP2	KIK(gl)IEDK
NET1	NESAQIDRQQK(gl)ETTSR	TOP2	TEEEENAPSSTSSSSIFDIK(gl)KEDK
NET1	SDLFK(gl)MIEGDDTDLPQWFK	TOP2	TPSVSETK(gl)TEEEENAPSSTSSSSIFDIKKEDK
NET1	SQAEPSGIVEPK(gl)R	TRI1	EIK(gl)LENESLPNLSG
NET1	VADLK(gl)SANIGGEDLNK	TRI1	HLFNPDEIVK(gl)HEEEQKQTPEK
NFI1	NENQGTVK(gl)QEQDYDSR	TRI1	VIIPK(gl)NDIISRDQEISIR
NHP10	KISNIDADDDKEENEQK(gl)IK	TRI1	VLLSAPLQK(gl)FLGSEELPR
NHP10	VADSK(gl)GGEDGSIVSSN	TRX1	FSEQYPQADFYK(gl)LDVDELGDVAQK
NOP12	LLNEEAEAEDDK(gl)PTVTK	TUP1	APESTLK(gl)ETEPENNNTSK
NOP12	SSAIDNIFGNIDEK(gl)KIESSVDK	TUP1	DAYEEEIK(gl)HLK
NOP56	PTLK(gl)NELAIQEAMELYNK	TUP1	DYDFK(gl)MNQQLAEMQQIR
NOP7	LDPTEIEEDVK(gl)VESLDASTLK	TUP1	ETTTLPSVK(gl)APESTLK
NSR1	LSWSIDDEWLK(gl)K	TUP1	INDTGSATTATTTTATETEIK(gl)PK(gl)EEDATPASLHQDHY LVPYNQR
NTG1	IK(gl)QEEVVPQPVDIDWVK	TUP1	IWNIQNANNK(gl)SDSK
NTG1	LENDISVK(gl)VED	TUP1	LQNQK(gl)DYDFK
NTG1	RPLVK(gl)TETGPESELLPEK	TYE7	K(gl)QDEDGAETAATTPIPSAAATSTK
NUM1	ESLSDK(gl)IEELTNQKK	TYE7	LQQIIPWVASEQTAFEVGDSVK(gl)K
PAA1	ELIK(gl)EEYDN	TYE7	SSETTLIK(gl)PESEFDNWLSDENDGASHINVNK
PDC1	LTQDK(gl)SFNDNSK	TYE7	TNIDAK(gl)ETK
PDR1	TSLEGTTVQVK(gl)EETDSSSTSFSNPQR	UBA2	IK(gl)QETNELYELQK
PGI1	TLSVK(gl)QEFQK	UBA2	LLAIENLWK(gl)TR
PGK1	VK(gl)ASKEDVQK	UBA2	SHIFNIPMK(gl)SVFDIK
PGK1	VLENTEIGDSIFDK(gl)AGAEIVPK	UBC9	EGTNWAGGVYPITVEYPNEYPSK(gl)PPKVK
POB3	KEESSNEVVPK(gl)KEDGAEGEDVQMAVEEK	UBC9	VLLQAK(gl)QYSK
POL30	DLSQLSDSINIMITK(gl)ETIK	UBI1	LIFAGK(gl)QLEDGR
POL30	LMDIDADFLK(gl)IEELQYDSTLSLPSSEFSK	UBI1	TLSDYNIQK(gl)ESTLHLVLR
PRE2	VK(gl)EEEGSFNNVIG	UBI4	LIFAGK(gl)QLEDGR
PRP45	DVSEK(gl)IILGAAK	UBI4	TLSDYNIQK(gl)ESTLHLVLR
PRP45	K(gl)QTSTVAR	UME1	STIDIAEDNKIK(gl)NEEFK
PRP45	LDEAVNVK(gl)SEGASGSHGPIQFTK	UME6	DREITDPNVK(gl)LDENESK
PTA1	KIK(gl)METEPLAEEPEEPEDDDRMQK	UPC2	ADGSVESDSSVDLPPTIK(gl)K
PUS1	K(gl)ADFDDEK(gl)DKK	UTP7	TNSDIPDVK(gl)PDVK
RAD16	NDNDEIIEIK(gl)EER	VBA5	AENK(gl)GIIQQIK
RAD52	K(gl)PVFGNHSEDIQTKLDK	VHR1	NLFNIINK(gl)NK
RAD52	NLVK(gl)IENTVSR	VHR2	LQK(gl)FDIEDQPLESEQEYDFIAK
RAD59	NEANTNYNLLSATNSKPTFIK(gl)LEDAK	VIP1	SGIK(gl)KEPIESDEVPQQETK
RAP1	DSIRPK(gl)TEIISTNTNGATEDSTSEK	VMA1	AIK(gl)EESQSIYIPR
RBA50	DVHFIK(gl)EESQNEINIEKIDINDPNFNDK	VPS3	K(gl)TEDDSLR
REB1	AIIDADSITQHPDFQQYINTAADTDDNEK(gl)IK	VPS72	SDIK(gl)RDETTNEDSDDQVR
REB1	ELVDYFSSNISMK(gl)TEN	VPS72	VNSDELK(gl)PTALPDVTLDAIANK
REH1	KGMK(gl)KMQQIEK	YAP5	QK(gl)LETLTLK
REP2	GAYK(gl)LQNTITEGPK	YJR129C	IK(gl)IEETPNLISAASTTGFR
REP2	MDDIETAK(gl)NITVK	YLR455W	NSISIK(gl)EDPEDNQK
REP2	NLTVK(gl)AR	YMR111C	IK(gl)PEPGLSDFENGEYDGNESDENATTR
RHR2	TYDAIAK(gl)FAPDFADEEYVNKLEGEIPEK	YRR1	YLK(gl)LTR
RLP7	SSTQDSK(gl)AQTINSNPEIIIRK	YSH1	IEPIK(gl)EENEDNLDSQAEK
RNR2	DSK(gl)SNLNK	YTA7	VGYETQIK(gl)DENGIIHTTTR
RNR2	STK(gl)QEAGAFTFNEDF	ZEO1	AETAAQDVQQK(gl)LEETK
RPA34	VEGLK(gl)LEHFATGYDAEDFHVAEEVK	ZEO1	GQEVK(gl)EQAEASIDNIK
RPB4	HLK(gl)HENANDETTAVEDEDDDLDEDDVNADDDDFMH-SETREK	ZEO1	NEATPEAEQVK(gl)K
RPC37	SEEVK(gl)AEDDTGEEEEDDPVIEEFPLK		

### Verification of novel SUMO substrates

**Figure 3:**
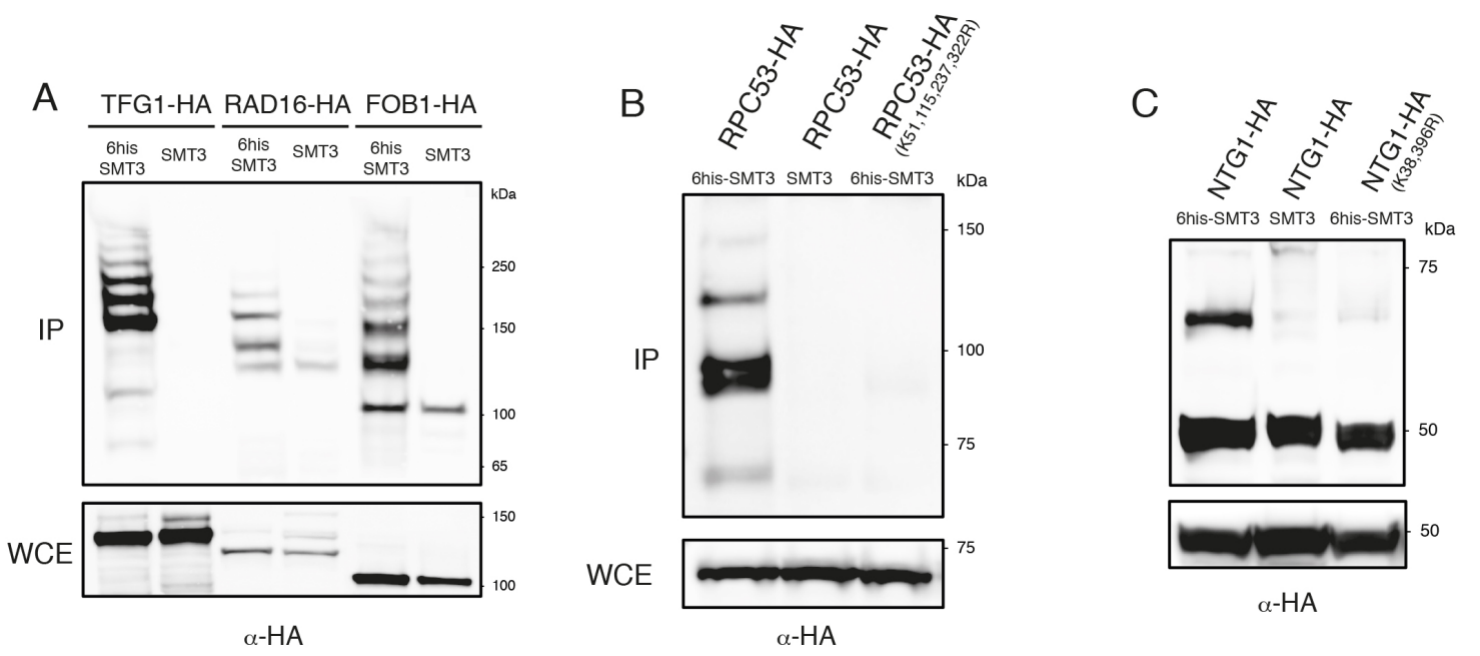
FIGURE 3: Mutational analysis of SUMO sites identified. **(A)** Histidine pulldowns from cells carrying *TFG1*, *RAD16* and *FOB1* tagged with 3 HA epitopes in strains expressing *SMT3* tagged with 6 histidines (*6his-SMT3*) or wild-type *SMT3* (untagged control). Western blot analysis using α-HA antibodies in the pulldown demonstrates the presence of SUMO conjugates for *TFG1*, *RAD16* and *FOB1*. **(B)** Histidine pulldowns from cells carrying *RPC53 or RPC53 (K38,115,237,322R)* tagged with 3 HA epitopes. The analysis demonstrates that lysines 38, 115, 237 and 322 (identified in our screen) are sites for the conjugation of *SMT3* in *RPC53*. **(C)** Histidine pulldowns from cells carrying *NTG1 or NTG1 (K38, 396R) *tagged with 3HA epitopes. The analysis demonstrates that lysines 38 and 396 (identified in our screen) are sites for the conjugation of *SMT3* in *NTG1*.

To confirm that some of the yeast proteins newly identified as potential SUMO substrates are indeed modified by SUMO we tagged the proteins of interest with either Myc- or HA-epitopes in strains where Smt3 contains a HIS/Flag tag (HF-Smt3) at its N-terminus. Cells were grown and lysed under denaturing conditions and Smt3 was purified using Ni-nitrilotriacetic acid (Ni-NTA) beads. The immunoprecipitates were separated by SDS-PAGE, and the different forms of the tagged proteins were resolved by immunoblotting against the epitope tags. It is important to note that immunoprecipitation of SMT3 using histidine tags allows us to work under denaturing conditions, which significantly reduces loss of SUMO conjugation in the lysates due to endogenous SUMO proteases. We confirmed the SUMOylation of several newly identified substrates, including the transcription factor Tfg1, the nucleotide excision repair (NER) factor Rad16, the replication fork barrier protein Fob1 (Fig. 3A), the RNA Polymerase III subunit Rpc53 (Fig. 3B) and the base excision repair protein Ntg1 (Fig. 3C). Multiple SUMOylated species were observed for Tfg1, Rad16 and Fob1 (Fig. 3A).

### Mutational analysis of SUMO-acceptor sites identified by mass spectrometry

Following the confirmation that we had identified novel SUMO-substrates during our proteomic analysis, we proceeded to test whether mutation of the lysine residues identified as potential conjugation sites prevented SUMOylation of these substrates. We identified four potential conjugation sites on Rpc53 (K51, 115, 237, 322) and two on Ntg1 (K38, 396). Strains carrying a tagged wild-type copy or a mutant allele where all acceptor lysines identified by mass spectrometry had been substituted for arginine were analyzed for Rpc53 and Ntg1. SUMOylated forms were detected in the immunoprecipitates for all the proteins when Smt3 was tagged (Fig. 3B-C), however SUMO forms were absent when the SUMO-acceptor lysines identified by mass spectrometry were mutated to arginine (Fig. 3B-C), thus these lysines represent bona fide SUMO-acceptor lysines in the target proteins and validate the success of our approach.

## DISCUSSION

Identifying lysine residues where SUMO is conjugated to substrates is a challenging process that in many cases is resolved by site-directed mutagenesis of potential acceptor lysines. This approach is not only labour intensive but often unable to discriminate between bona fide conjugation sites and lysines that alter the protein biology in a manner that leads to reduced SUMOylation levels (at other domains of the protein). Recent studies in mammalian cells have bypassed the challenges of spectrometric analysis of protein SUMOylation using differential protease cleavage of a modified SUMO2 lacking lysine residues 12. Here we have employed a similar approach to map acceptor lysines for SUMO in endogenous substrate proteins purified from yeast cells under several conditions. Our analysis revealed a total of 257 SUMO-acceptor lysines. Over 70% of the sites identified adhere to previously described SUMO motifs, including the core consensus motif ([VILP]KxE) 22, the phosphorylation-dependent motif ([VILP]Kx[ED]x[S or xS]) 10, the negatively charged/acidic consensus motif ([VILP]Kx[ED][ED][ED]) 11 and the inverse consensus motif ([ED]xK[VILP]) 12.

SUMOylation substrates were found in all major cellular compartments. However, the presence of SUMOylated proteins was lower in the ER, Golgi’s apparatus, mitochondrion, membrane and cytoplasm when proportionally compared with the whole proteome. On the other hand, the amount of SUMOylated proteins is proportionally higher in ribosomes and much higher in the nucleus. A closer look at nuclear proteins shows that a high proportion of SUMOylated proteins is found in association with chromatin (i.e. transcription factors) (Fig. S2). Functionally, SUMOylated proteins were involved in a wide range of biological processes. There were a large number of SUMOylated proteins involved in nuclear processes such as chromatin organization, transcription and DNA damage. A few SUMO-acceptor lysines were found on proteins from the ubiquitination and protein degradation pathway, including ubiquitin itself. This raises the possibility that SUMOylation of ubiquitin might affect the ubiquitination pathway directly.

SUMOylation is known to play an important role in the metabolism of the chromatin and in gene expression. We identified a large number of SUMO-acceptor lysines on nuclear proteins. Most of these proteins are related to transcription and chromatin organization. In accordance with previous studies we found SUMO-acceptor lysines on components of the general transcription machinery as well as on components of gene-specific transcription pathways. SUMO-acceptor lysines were identified in six subunits of RNA polymerases and on components of the TFIID, TFIIF and TFIIIB, including the TATA-binding protein (TBP). Other components of the general transcription found SUMOylated included the transcription repressors Ncb2, Tup1 and Cyc8. SUMOylated proteins related to gene-specific transcription include the transcription factors Crz1 and Sko1 (stress response genes), Gzf3 (of nitrogen catabolic genes), and Cin5 (drug response genes).

Control of chromatin function is likely to involve not only SUMOylation of core histones but also proteins involved in the modification and exchange of histones. We found SUMO-acceptor lysines on histone acetylases (i.e. Gcn5), and on components of the RPD3 complex (involved in histone deacetylation), on the Swr1 complex (involved in histone exchange) and of the COMPASS complex (involved in histone methylation). In addition, we found SUMOylation on various chromatin remodelling complexes.

SUMOylation has also been linked to centromeric function. One of the key SUMO-substrate in this regulatory function is Top2, which controls local chromatin structures in the centromeric region. We mapped SUMO-acceptor lysines not only on Top2 but also on other kinetochore proteins like Cbf2, and on components of other centromeric complexes (CPC complex, Ndc80 or Dam1 complexes). These complexes are important players in the regulation of chromosome segregation and kinetochore clustering. The detailed functional significance of Top2 SUMOylation and of SUMOylation of other components with centromeric functions and in chromosome segregation pathways remains unclear. We hope that the SUMO-acceptor dataset presented here will be useful for future functional studies of SUMOylation in yeast.

## MATERIALS AND METHODS

### Yeast strains

Strains used in this study are isogenic to BY4741 (*MATa his3*∆*1 leu2*∆*0 met15*∆*0 ura3*∆*0*), W303 (*Mata can1-100 leu2-3 his3-11 trp1-1 ura3-1 ade2-1*) or DF5a (*MATa his3*∆*200 leu2-3 lys2-801 trp1-1 ura3-52).* Strains used are listed in Supplemental Table S1.

### Yeast media and cell cycle synchronizations 

Cells were grown on complete media YPED in broth or solid form (3% yeast extract, 1% peptone, 1% glucose/dextrose, 2% agar for solid media). For plates containing MMS (in DMSO), the genotoxin was added to warm YPD.

### Drops analysis by growth tests

10-fold dilutions of fresh cells were made in PBS. Cells were spotted (as 2 - 5 µl drops) onto solid media, incubated at the appropriate temperature for 3 - 5 days and then photographed.

### Purification of His-tagged proteins 

For His-tag purifications 100 OD of cells were harvested (4000 rpm, 2 minutes), washed once with water, and the cell pellet frozen at -80°C. The cell pellet was resuspended in 500 µl Buffer A (8 M Urea, 100 mM NaH_2_PO_4_, 10 mM Tris HCl, 0.05% Tween pH 8), an equal volume of glass beads were added, and the cells lysed by 1 45s cycle, power 6 in a FastPrep FP120 (BIO 101) machine. Tubes were pierced with a hot needle and placed onto fresh eppendorfs and spun (1000 rpm, 2 minutes) to collect lysate minus glass beads. Cell lysate was clarified by centrifugation (14000 rpm, 15 minutes, 4°C) Protein concentration was determined using a Bradford Assay, and 15 mg of protein in 1ml was added to 50 µl of a 50:50 slurry of Ni-NTA beads (Qiagen) (prewashed in Buffer A). Imidazole was added to a final concentration of 20 mM, to reduce non-specific binding. Proteins were bound for 2 - 3 hours at 4°C on a rotating platform, before the beads were washed 3 times in Buffer A containing 2 mM imidazole, followed by 5 washes in Buffer B (8 M Urea, 100 mM NaH_2_PO_4_, 10 mM Tris HCl, 0.05% Tween pH 6.3). Bound proteins were eluted off the beads using 30 µl x2 NuPAGE loading buffer (Invitrogen) supplemented with 4% β-mercaptoethanol and 200 mM EDTA. Eluates were loaded onto 3-8% Tris-Acetate or 4-12% Bis-Tris pre-cast NuPAGE gels (Invitrogen) and analysed by western blotting.

### Sample preparation for western blot analysis

Samples were prepared by TCA extraction. Extracts were prepared as follows, cells were collected by centrifugation (4000 rpm, 2 minutes) and washed with 20% TCA. The TCA was aspirated and the pellets frozen at -80°C. All of the following purification steps were performed on ice with pre-chilled solutions. Cells were resuspended in 250 µl 20% TCA, glass beads were added and the cells broken by 1 40s cycle, power 5.5 in a FastPrep( FP120 (BIO 101) machine. Tubes were pierced with a hot needle and placed onto fresh eppendorfs and spun (1000 rpm, 2 minutes) to collect lysate minus glass beads. The glass beads were washed with 1 ml 5% TCA and this was added to the lysate, and mixed by pipetting. The precipitated proteins were collected by centrifugation (14000 rpm, 10 minutes 4(C) and then pellets were washed with 750 µl 100% ethanol. Proteins were solubilized in 50 µl 1M Tris pH 8 and 100 µl x2 SDS-PAGE loading buffer (60 mM Tris pH 6.8, 2% SDS, 10% glycerol, 0.2% bromophenol blue) and boiled for 5 minutes at 95°C. Insoluble material was removed by centrifugation (14000 rpm, 5 minutes, room temperature) and the supernatant either stored at -20°C or loaded immediately onto a SDS-PAGE mini-gel. Samples were either run on an 8% acrylamide gel in Tris-Glycine SDS running buffer using the Bio Rad Mini-PROTEAN 3 system or on Pre-Cast 4-12% Bis-Tris gels in NuPAGE MOPS (all Invitrogen). SDS-PAGE gels were transferred to polyvinylidene fluoride transfer membrane (Hybond-P, Amersham Biosciences) in either the Bio-Rad Mini Trans-Blot Electrophoretic Transfer Cell or by using the XCell SureLock Mini Cell Transfer module (Invitrogen). The Bio-Rad system was used in conjunction with Tris-Glycine blotting buffer (National Diagnostics) containing 20% methanol, and run for 1h at 200V or overnight at 30V. The NuPAGE system was used with NuPAGE transfer buffer containing 20% methanol and run for 1h at 30V.

### Immunological detection

Membranes were blocked in 5% skimmed milk powder in PBS with 0.1% Tween 20 (PBS-T) for 1h or overnight at 4°C, then incubated with either mouse monoclonal anti-c-myc IgG1κ antibody 9E10 (Roche) or anti-HA IgG1 antibody 12CA5 (Roche) in blocking solution for between 1 hour at room temperature to overnight at 4°C. Following several washes in PBS-T, membranes were incubated with the sheep anti-mouse IgG Horseradish Peroxidase-linked antibody (GE Healthcare) at a 1/10000 dilution in blocking solution. After several further washes in PBS-T, membranes were incubated with the ECL Plus Western Blotting Detection System (GE Healthcare) followed by exposure to ECL Hyperfilm (GE Healthcare), to detect the secondary antibody.

### Purification of SUMOylated proteins for mass spectrometry

Nine liters of culture harvested at O.D. 0.9 were used in each experiment. Cells were pelleted and washed in chilled PBS with 20 mM NEM. Cells were resuspended in 250 ml of lysis buffer (1.85 N NaOH, 1.85% β-mercaptoethanol) and incubated in ice for 30 minutes. TCA (trichloroacetic acid) was added to a final concentration of 25% and kept in ice for a further 30 minutes. Sample were spun at 15.000 rpm for 10 minutes in acetone-resistant tubes. The pellet washed with 250 ml acetone and spun again. The pellet was air-dried for 10 minutes and resuspended in 100 ml of binding buffer (6 M guanidine hydrochloride, 100 mM NaH_2_PO4, 10 mM Tris-HCl, 20 mM NEM, pH 8) by vortexing for 10 minutes at RT. Resuspended proteins were recovered after a spun at 15.000 rpm. for 10 minutes. All spins were performed at 4°C. Protein extraction after elution from agarose beads (see Purification of His-tagged proteins) was adjusted to pH 7 and filtered through a 50 kDa MWCO protein filter (Vivaspin 20, GE Healthcare) up to 1 ml (one filter was used for every 12 ml of elution sample). Buffer A (w/o Tween®-20) was added in a proportion 10:1 and the sample filter again up to 1 ml. Sample was placed in a 10 kDa MWCO protein filter (Vivaspin 2, GE Healthcare), and filtered up to a volume 1/100000 of the original volume of the culture (i.e. 90 µl from a 9 l culture). Protein sample was then digested with Lys-C endonuclease (Roche) at final concentration of 0.02 µg/µl. The reaction was incubated at 37°C for 12 hours. A standard 4% β-mercaptoethanol/2% SDS loading buffer was added to the sample, left on the bench for 15 minutes and loaded into a 12% Tris-Bis NuPAGE® gel (Invitrogen). Gel was run using an Invitrogen system with NuPAGE® MOPS SDS running buffer (Invitrogen) for 2 h at 140 V. After the running, the gel was stained using SimplyBlue™ SafeStain (Invitrogen) following manufacturer’s protocol. The band of interest was cut from the gel. Peptides were in-gel digested using trypsin. First, gel bands were destained using 40% acetonitrile, 200 mM ammonium bicarbonate. Gel pieces were shrunk with acetonitrile and enough trypsin solution (10 ng/μl trypsin in 10% acetonitrile in 50 mM ammonium bicarbonate) was added to cover the gel pieces. Gel pieces were incubated overnight at 37°C. Peptides were extracted with 5% formic acid in acetonitrile. The extracts were dried to completeness in a vacuum concentrator and resuspended in 10 μl of 0.1% trifluoric acetic acid (TFA). Peptide mixtures were loaded onto a 75 µm i.d. c18 column (pepmap, ThermoFisher) and eluted over a 60 minute gradient stretching from 3% acetonitrile to 50% acetonitrile (both in 0.1% formic acid) at 300 nl/minute using an RSLC Ultimate 3000 onto a LTQ-Orbitrap-Velos (both ThermoFisher). Profile spectra were acquired in the Orbitrap (60,000 resolution) and the 6 most intense ions excluding +1 charge state were selected for fragmentation in the linear ion trap (LTQ). Raw data was processed using MaxQuant and searched using the embedded Andromeda routine 14. Data was searched against a *Saccharomyces cerevisiae* database (to which the recombinant Smt3 was manually added) using default settings. Mass tolerances for Ms and MS/MS data were 10 part per million (ppm) and 0.5 Da respectively. Methionine oxidation and glygly modification of lysine were allowed as variable modification. The false discovery rates at the peptide, protein and site level were 0.005, 0.01 and 0.01 respectively. Statistical analysis and charts were created using the SPSS software environment. Graphic representation of the local context of the SUMO-acceptor lysines was created using iceLogo 23. SUMO motifs were assigned to sumo-acceptor lysines using the 3of5 web application for pattern matching 24. Subcellular distribution and function of SUMOylated proteins was obtained from the Gene Ontology (GO) slim mapper part of the *Saccharomyces* Genome Database (SGD, http://www.yeastgenome.org.

## SUPPLEMENTAL MATERIAL

Click here for supplemental data file.

All supplemental data for this article are also available online at http://microbialcell.com/researcharticles/identification-of-sumo-conjugation-sites-in-the-budding-yeast-proteome/.
