# Supplementary material for: Identification of SUMO conjugation sites in the budding yeast proteome
Source: Microb Cell. 2017 Oct 2;4(10):331–41. doi: 10.15698/mic2017.10.593 (PMC5657824; doi:10.15698/mic2017.10.593)
Supplement: Supplementary file 1 [file mic-04-331-s01.pdf]

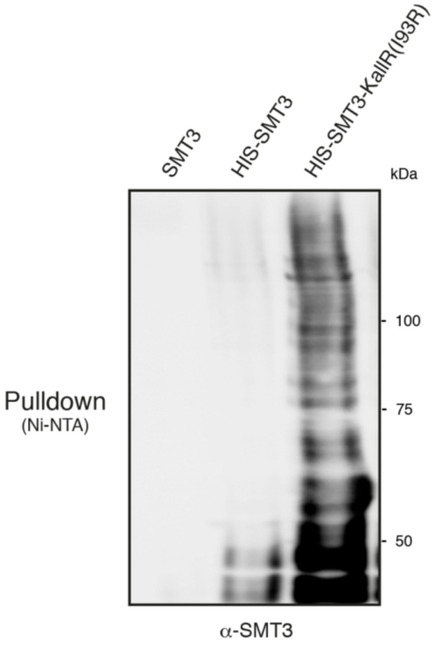

FIGURE S1: Smt3 immunoblotting of Smt3 in WT and Smt3 variants, His-SMT3 and His-SMT3-KallR-I93R). After HIS pull down samples were separated by SDSPAGE gel.

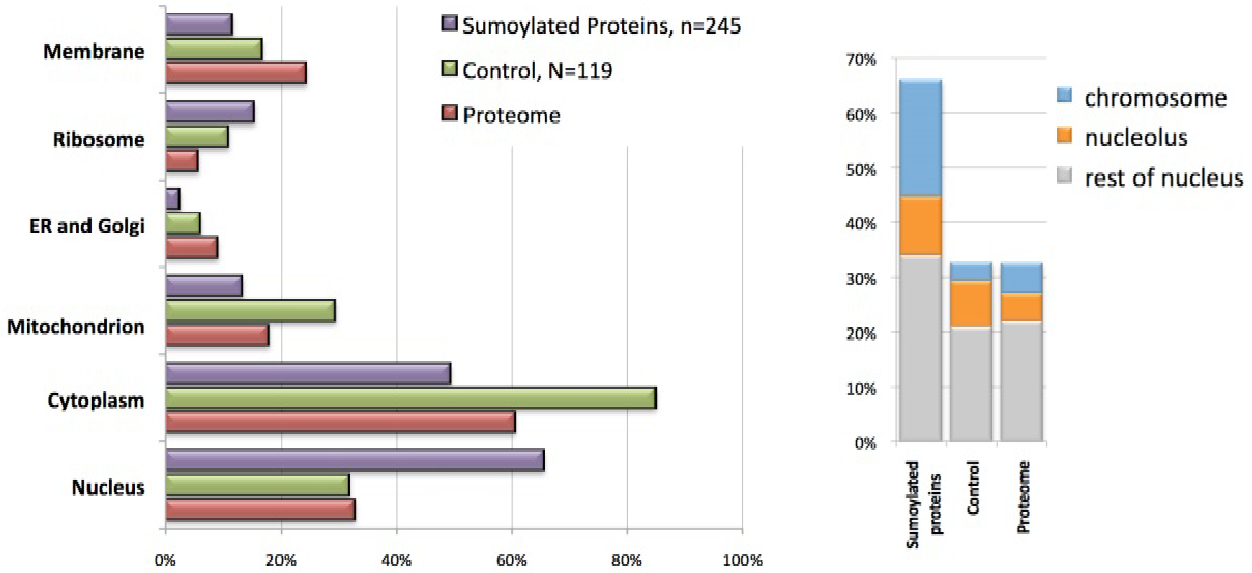

FIGURE S2: Subcellular distribution of the SUMOylated proteins detected by mass spectrometry. Subcellular distribution of SUMOylated proteins was obtained from the Gene Ontology (GO) slim mapper at the SGD website.

TABLE S1. Yeast strains used.

Strain	Genotype
BY4741	*MATa his3Δ1 leu2Δ0 met15Δ0 ura3Δ0*
CCG4620	*MATa his3Δ1 leu2Δ0 met15Δ0 ura3Δ0 6HisFLAG-smt3::kanMX6*
CCG9474	*MATa, trp1-1, ura3-52, his2Δ200, leu2-3, 112, lys2-801 Smt3ΔHIS [6HIS-SMT3 –KallR-I93R LEU]*
CCG10036	*MATa his3Δ1 leu2Δ0 met15Δ0 ura3Δ0 6HisFLAG-smt3::kanMX6 pRS415 RPC53-5HA-KtoR*
CCG10030	*MATa his3Δ1 leu2Δ0 met15Δ0 ura3Δ0 6HisFLAG-smt3::kanMX6 pRS415 NTG1-5HA*
CCG10032	*MATa his3Δ1 leu2Δ0 met15Δ0 ura3Δ0 6HisFLAG-smt3::kanMX6 pRS415 NTG1-5HA-KtoR*
CCG9311	*MATa his3Δ1 leu2Δ0 met15Δ0 ura3Δ0 Rad16-9Myc HYG*
CCG9314	*MATa his3Δ1 leu2Δ0 met15Δ0 ura3Δ0 Rpc53-9Myc HYG*
CCG10050	*MATa his3Δ1 leu2Δ0 met15Δ0 ura3Δ0 pRS415 NTG1-5HA*
CCG10054	*MATa his3Δ1 leu2Δ0 met15Δ0 ura3Δ0 pRS415 RPC53-5HA*
CCG7565	*MATa his3Δ1 leu2Δ0 met15Δ0 ura3Δ0 6HisFLAG-smt3::kanMX6;Fob1D NAT; pRS406-Fob1-12HA-WT URA*
CCG9368	*MATa his3Δ1 leu2Δ0 met15Δ0 ura3Δ0 6HisFLAG-smt3::kanMX6; Tfg1-9Myc HYG*
CCG9369	*MATa his3Δ1 leu2Δ0 met15Δ0 ura3Δ0 6HisFLAG-smt3::kanMX6; Rad16-9Myc HYG*
CCG9368	*MATa his3Δ1 leu2Δ0 met15Δ0 ura3Δ0 6HisFLAG-smt3::kanMX6; Tfg1-9Myc HYG*
CCG9369	*MATa his3Δ1 leu2Δ0 met15Δ0 ura3Δ0 6HisFLAG-smt3::kanMX6; Rad16-9Myc HYG*
CCG10034	*MATa his3Δ1 leu2Δ0 met15Δ0 ura3Δ0 6HisFLAG-smt3::kanMX6 pRS415 RPC53-5HA*

TABLE S2.

Gene name	Prob	PEP	GlyGly (gl) site	GlyGly (K) Probabilities	Charge
SIZ1	1.00	0.000270814	NLMNDNDDDDDDRLMAEITSNHLK**(gl)**STNTDILTEK(x)	K(0.997),K(0.003)	5
GAL3	1.00	0.00917997	LTGAGWGGCTIHLVPSGANGNVEQVRK**(gl)**ALIEK(x)	K(1),K(1)	4
STI1	0.92	0.0122443	APQK**(gl)**EESK**(gl)**ESEPMEVDEDDSK**(gl)**IEADK(x)	K(0.079),K(0.92), K(0.284),K(0.717)	4
ENO2	1.00	0.00088398	K**(gl)**RYGASAGNVGDEGGVAPNIQTAEEALDLIVDAIK	K(1)	4
SCY1	0.99	0.00538374	VIEPTIMK**(gl)**K**(gl)**EDPETVAAK**(gl)**NIEVAAMQPVK(x)	K(0.989),K(0.989), K(0.075),K(0.946)	4
TOP2	0.59	0.0023933	TEEEENAPSSTSSSSIFDIK**(gl)**K**(gl)**EDK**(gl)**DEGELSK(x)	K(0.075),K(0.027), K(0.304),K(0.593)	5
ISC1	1.00	0.0148749	FFRGLHFWASILLLIASLVVTTFTANK(x)	K(1)	3
RPA49	1.00	0.00609187	MSVKRSVSEIEIESVQDQPSVAVGSFFK(x)	K(1)	4
ESC1	0.94	0.00109534	VNEGEEPEHQAVDIPVK**(gl)**VEVK**(gl)**EEQEEMPSK	K(0.935),K(0.064)	5
RPC53	0.76	0.011834	LPAFERPAVK**(gl)**EEK**(gl)**EDMETQASDPSK(x)	K(0.241),K(0.757), K(0.002)	4
SPC24	0.72	0.00448937	INVAKLEGDLEYTNEESNEFGSK**(gl)**DELVK(x)	K(0.715),K(0.285)	4
PCC1	1.00	0.0044897	QATIATK**(gl)**VLSPDPILKPQDFQVDYSSEK	K(1)	4
MTR4	1.00	0.00502438	GIVIMMIDEK**(gl)**MEPQVAK**(gl)**GMVK(x)	K(1),K(1),K(1)	3
PCK1	0.50	0.0117074	AMEMIILGTEYAGEMK**(gl)**K(x)	K(0.5),K(0.5)	3
RPA34	0.93	0.00120941	K**(gl)**DVPK**(gl)**VEGLK**(gl)**LEHFATGYDAEDFHVAEEVK	K(0.019),K(0.046), K(0.935)	5
YLR278C	1.00	0.000163509	YSSSTSNSNTNNNTPTAGTVPPTPHPVIK**(gl)**R	K(1)	4
SLK19	0.98	0.00217688	FIINDGVERNDSFNINTDTLK**(gl)**LENDINEK(x)	K(0.979),K(0.021)	4
GYP7	1.00	0.0163428	AELLFKKFEK**(gl)**MMHVMER	K(1)	3
BUD3	0.43	0.00456219	TGNEDVGNNNPSNSIPK**(gl)**IEK**(gl)**PPAFK(x)	K(0.149),K(0.425), K(0.425)	4
MOT1	0.84	6.62807E-10	AVGGIVAHAPSWDPNESDLVGGTNEGSPLDNAQVK**(gl)**L EHEMK(x)	K(0.844),K(0.156)	5
RPN6	1.00	0.00573342	YMLLSKIMLNLIDDVK**(gl)**NILNAK(x)	K(1),K(1)	3
FHL1	1.00	4.45176E-10	HPQNTTTDIENEVENPVTDDNGNLK**(gl)**LELPDNLDNADF SK	K(1)	4
PCK1	0.85	0.00376625	AMEMIILGTEYAGEMK**(gl)**K(x)	K(0.848),K(0.152)	3
SKY1	1.00	0.0107315	K**(gl)**NFFQRDYNMMKK	K(1)	3
IMA5	1.00	0.00237767	EGELNMMFNFK**(gl)**HTSVGENPK(x)	K(1),K(1)	2
FAT1	1.00	0.01485	YK**(gl)**EDWYIIPYFLK(x)	K(1),K(1)	3
RPO26	1.00	0.000948782	DGETTDANGK**(gl)**TIVTGGNGPEDFQQHEQIR	K(1)	4
YDL156W	1.00	0.0063267	LRGESADDVK**(gl)**GIPNVNDNQLLK	K(1)	4
NAM2	1.00	0.00936169	KSIMGMLNSEGLSK**(gl)**SVVR	K(1)	2
RPC37	0.50	0.0115737	SHLWEIDIPLDEQAFYNK**(gl)**DK(x)	K(0.5),K(0.5)	4
AIM22	0.57	0.0102356	FETKFFNK**(gl)**MIIK(x)	K(0.427),K(0.573)	3
PRP45	0.93	0.00725935	SEGASGSHGPIQFTK**(gl)**AESDDK(x)	K(0.934),K(0.066)	4

SPA2	1.00	0.000959672	TIK(gl)REEEDEDFDRVNHNIQITGAYTK	K(1)	5
MRD1	1.00	0.000430478	LK**(gl)**REEEDSSVQGNSLLAHALALK	K(1)	4
CYC8	0.50	0.00015421	QPTHAIPTQAPATGITNAEPQVK**(gl)**K(x)	K(0.5),K(0.5)	4
YKU80	0.98	0.0144462	LKLDSELK**(gl)**TELEREK(x)	K(0.979),K(0.02)	4
REB1	0.50	2.00374E-05	AIIDADSITQHPDFQQYLNTAADTDDNEK**(gl)**LK(x)	K(0.5),K(0.5)	4
HIR2	1.00	0.00429266	SFPENIK**(gl)**LEESASAAPIPNDIGR	K(1)	3
TAF8	1.00	0.00150065	TK**(gl)**REQDEGHDLELLLNNEHAR	K(1)	5
SUM1	1.00	1.88416E-06	RYFVEPSTK**(gl)**QESLLLSAPSSSRDDADMSLTSVPQR	K(1)	4
MLP1	0.56	0.0134363	K**(gl)**IK**(gl)**TEDEEEKETDK	K(0.558),K(0.442)	4
PGK1	1.00	0.0049469	VLENTEIGDSIFDK**(gl)**AGAEIVPK(x)	K(0.996),K(0.004)	3
SWI3	1.00	0.000798961	IQKEEEPENNTVIEGVK**(gl)**EESQPDENTK(x)	K(0.999),K(0.001)	4
BDP1	0.54	0.00102036	ARQEFK**(gl)**PLHSLTK**(gl)**EEQEEEEEK	K(0.537),K(0.463)	5
SPT7	1.00	7.29666E-05	NGFGTVLK**(gl)**QEDDDQLQFHNDHSLNGNEAFEK	K(1)	4
PET54	1.00	0.00276996	ELLTLSEK**(gl)**GDAADYSLEMDDSK(x)	K(1),K(1)	3
BOI2	0.99	0.00824908	RMEDELDMK**(gl)**PGDK(x)	K(0.992),K(0.008)	2
SLI15	1.00	0.000907482	RFDNQTWAAK**(gl)**EEMENEPILQALK	K(1)	4
IFH1	0.80	8.82816E-09	FK**(gl)**K**(gl)**EDDGISFGNGNEGYNEDIGEEVLDLK	K(0.798),K(0.202)	3
SOD1	1.00	5.68654E-07	GDAGVSGVVK**(gl)**FEQASESEPTTVSYEIAGNSPNAER	K(1)	3
TOP2	0.67	0.00269652	TEEEENAPSSTSSSSIFDIK**(gl)**K**(gl)**EDK(x)	K(0.165),K(0.165),K(0.669)	3
BRP1	1.00	0.00324955	HMDNILK**(gl)**MSIPVK(x)	K(1),K(1)	3
VMA10	1.00	0.0123066	IQK**(gl)**DK**(gl)**ELK**(gl)**EFEQK(x)	K(1),K(0.996),K(0.032), K(0.972)	2
VPS72	0.99	0.000443579	ESEESIK**(gl)**NDGDVNSLGENSSSVHNQK(x)	K(0.99),K(0.01)	4
AIM41	1.00	0.00377516	K**(gl)**AMIAK**(gl)**DEMK**(gl)**K(x)	K(1),K(0.975),K(0.048), K(0.977)	2
RPS0B	1.00	0.0154587	AVLK**(gl)**FAAHTGATPIAGR	K(1)	3
SUM1	1.00	0.00112412	ERPSTANSSSITPTPVTPNNLIQIK**(gl)**R	K(1)	4
SNF2	1.00	0.00254668	TK**(gl)**K**(gl)**EDK**(gl)**SEAIDGNGEIK(x)	K(1),K(1),K(1),K(1)	3
POB3	0.58	7.31909E-05	K**(gl)**EESSNEVVPK**(gl)**K**(gl)**EDGAEGEDVQMAVEEK	K(0.007),K(0.417),K(0.576)	4
MPP10	1.00	0.00182487	VK**(gl)**LDLFADEEDEPNAEGVGEASDK	K(1)	3
ABF1	1.00	0.00139347	RQHLSDITLEERNEDDK**(gl)**LPHEVAEQLR	K(1)	6
BUD3	0.97	0.00190931	TGNEDVGNNNPSNSIPK**(gl)**IEK**(gl)**PPAFK	K(0.974),K(0.026)	3
AOS1	1.00	0.0153617	VEK(gl)LSEDEIALYDR	K(1)	3
USO1	1.00	0.00683859	K(gl)SDEK(gl)LEQSK(gl)K(x)	K(1),K(1),K(1),K(1)	2
TFC3	0.77	0.00558719	TTVVVENTK(gl)EDK(x)	K(0.771),K(0.229)	3
RPL16B	0.99	0.0155991	YEDVVAK(gl)LEDK(x)	K(0.986),K(0.014)	3
LOC1	1.00	0.00923104	QDK(gl)LEEK(gl)K(gl)DEIK	K(0.999),K(0.975),K(0.026)	2
CPR1	1.00	0.0158426	VVFK(gl)LYNDIVPK	K(1)	3
STB3	1.00	9.76916E-05	EVSPPQAISVK(gl)SEASSSIFSK	K(1)	3
RPD3	0.96	0.00172093	DAEDLGDVEEDSAEAK(gl)DTK(x)	K(0.96),K(0.04)	3
RPL13B	0.95	0.0170783	APTVK(gl)YNRK(x)	K(0.951),K(0.049)	2

NET1	1.00	0.0125056	ITSGMLK**(gl)**IPEPR	K(1)	3
LAS17	1.00	0.0016372	MGLLNSSDK**(gl)**EIIK(x)	K(1),K(1)	2
CDC3	1.00	0.00962949	GQVLPDQPEIK**(gl)**FIR	K(1)	3
RFA1	1.00	1.28145E-05	NANFITLK**(gl)**QEPGMGGQSAASLTK	K(1)	3
TEF1	1.00	0.00151869	LPLQDVYK**(gl)**IGGIGTVPVGR	K(1)	3
YTA7	1.00	0.00146212	VGYETQIK**(gl)**DENGIIHTTTR	K(1)	3
BDP1	0.99	0.000693274	LLNADIPESDRK**(gl)**AHTAIQLK(x)	K(0.987),K(0.013)	3
KAP123	1.00	0.0124806	TSLLQTAFSEPK**(gl)**ENVR	K(1)	3
RSC2	1.00	1.08043E-07	TSVK**(gl)**RESEPGTDTNNDEDYEATDMDIDNPK	K(1)	4
YKR017C	0.97	0.00587088	TTALMECGRELLCK**(gl)**GICK(x)	K(0.031),K(0.969)	3
RPC37	1.00	0.000683123	SIDNK**(gl)**LFVTEEDEEDRTQDR	K(1)	3
NET1	1.00	0.0131688	EKEDTNDK**(gl)**LLEK	K(1)	3
UBP7	1.00	0.0139871	LSNSLSMLFNK(x)	K(1)	2
VPS63	1.00	0.0148586	GKSQPK**(gl)**R	K(1)	2
FLP1	1.00	1.5582E-06	EMIALK**(gl)**DETNPIEEWQHIEQLK	K(1)	4
SPP41	0.50	0.00395066	IATDLNEDASLSDK**(gl)**K(x)	K(0.5),K(0.5)	3
CBF2	0.77	7.30327E-08	FIRDNQPIK**(gl)**K**(gl)**EENIVNEDGPNTSR	K(0.768),K(0.232)	4
HSC82	0.97	0.00396844	K**(gl)**VK**(gl)**EEVQELEELNK	K(0.028),K(0.972)	3
SPP41	0.77	0.000136325	IATDLNEDASLSDK**(gl)**K**(gl)**DGDEK(x)	K(0.215),K(0.768),K(0.017)	4
YUR1	1.00	0.00905648	WWKNGSGK**(gl)**YFLK	K(1)	2
PTA1	0.84	3.09359E-09	K**(gl)**IK**(gl)**METEPLAEEPEEPEDDDRMQK	K(0.843),K(0.157)	4
SUM1	1.00	2.50245E-09	K**(gl)**TPGDEETTTFVPLENSQPSDTIRK	K(1)	4
RPC82	0.55	0.000360346	K**(gl)**LK**(gl)**TEDGFVIPALPAAVSK	K(0.555),K(0.445)	4
NET1	0.89	0.0021893	MIEGDDTDLPQWFK(gl)GK(x)	K(0.888),K(0.112)	3
MCD1	1.00	0.00301107	ELSEEK(gl)EVIFTDVLK	K(1)	3
ZEO1	0.50	1.30486E-08	NEATPEAEQVK(gl)K(gl)EEQNIADGVEQK	K(0.5),K(0.5)	4
IRC5	1.00	0.00397182	LK(gl)K(gl)TMFK(gl)ELIK	K(1),K(1),K(1)	2
TOP1	0.90	0.00291001	EELLPESQLK(gl)EWLEK(x)	K(0.903),K(0.097)	3
ENO2	1.00	2.10136E-06	IEEELGDK(gl)AVYAGENFHHGDKL	K(1)	4
NET1	1.00	0.000122522	RMTNFLDDNQVREK(gl)EDTNDK(x)	K(0.997),K(0.003)	4
MET28	1.00	3.05772E-08	VAATTAVVVK(gl)EEEAPVSTSNELDK	K(1)	3
ZEO1	1.00	0.00300764	AETAAQDVQQK(gl)LEETK(x)	K(0.995),K(0.005)	3
RPS3	1.00	7.031E-08	ALPDAVTIIEPK(gl)EEEPILAPSVK(x)	K(0.003),K(0.997)	3
YJR129C	1.00	0.000818773	IK(gl)IEETPNLISAASTTGFR	K(1)	3
RPL28	1.00	0.00747815	IPNVPVIVK(gl)AR	K(1)	3
NSR1	0.50	0.00367581	LSWSIDDEWLK(gl)K(x)	K(0.5),K(0.5)	3
USO1	1.00	0.016465	LAK(gl)ELDNLK(x)	K(1),K(1)	3
BRE1	0.51	2.68242E-08	K(gl)IK(gl)LELSDPSEPLTQSDVIAFQK	K(0.51),K(0.49)	4
HHF1	1.00	0.00795425	K(gl)ILRDNIQGITKPAIR	K(1)	3
VPS3	0.50	0.0118938	K(gl)K(gl)TEDDSLR	K(0.5),K(0.5)	2
VPS3	1.00	0.0118938	K(gl)TEDDSLR	K(1)	3
RPS12	1.00	0.00359605	VADAK(gl)QLGEWAGLGK	K(1)	3

SIZ1	1.00	1.46708E-07	VIPEYLGNSSSYIGK**(gl)**QLPNILGK	K(1)	3
BUD4	0.98	0.00389346	LLESDTK**(gl)**DDADLEK(x)	K(0.983),K(0.017)	3
RPO21	1.00	2.57616E-21	VDLLNTDHTLDPSLLESGSEILGDLK**(gl)**LQVLLDEEYK	K(1)	4
KRS1	1.00	0.0107106	MSQQDNVK(x)	K(1)	2
BDP1	0.50	0.00610443	TEVVLGTIDDLK**(gl)**RK(x)	K(0.5),K(0.5)	3
SWR1	1.00	0.00114745	LLAQAEDEDDVK**(gl)**AANLAMR	K(1)	3
TOP2	1.00	0.01328	ILLEQK**(gl)**LVTK	K(1)	3
HHT1	1.00	0.01328	FQK**(gl)**STELLIR	K(1)	3
NET1	0.86	0.00249893	NEIDLDDSAPVSLYK**(gl)**SVK(x)	K(0.143),K(0.857)	3
BSP1	1.00	0.00324168	PASFLSSLEDNK**(gl)**LTK(x)	K(1),K(1)	3
PRP45	0.92	0.00418544	YHNGTPQTGAIVK**(gl)**PK(x)	K(0.92),K(0.08)	3
NET1	1.00	0.00226504	SQAEPSGIVEPK**(gl)**R	K(1)	3
HSP42	1.00	0.00232661	SEAPK**(gl)**EEAGETNK	K(1)	3
GPM1	1.00	0.00459396	KVYPDVLYTSK**(gl)**LSR	K(1)	3
SPP41	0.96	0.00584276	LIDVSLKPLNEAK**(gl)**PK(x)	K(0.961),K(0.039)	4
PRE2	1.00	0.00795468	VK(gl)EEEGSFNNVIG	K(1)	2
SSD1	1.00	0.00371963	KVNSTVAEK(gl)IYTK	K(1)	2
STH1	1.00	0.00268963	LIQLDELPK(gl)VFR	K(1)	3
YER064C	1.00	1.23162E-05	LQK(gl)FDIEDQPLESEQEYDFIAK	K(1)	3
SWR1	1.00	0.00774887	KGDIELK(gl)LESIAPLVR	K(1)	3
SLI15	0.99	0.000764443	NNVYMNTLK(gl)YEDK(x)	K(0.991),K(0.009)	3
NGG1	1.00	0.00253185	NPK(gl)SEFVVSQTLPR	K(1)	3
NHP10	0.75	0.000471333	KISNIDADDDK(gl)EENEQK(gl)IK(x)	K(0.107),K(0.752),K(0.141)	4
HSC82	0.98	0.0035121	K(gl)PK(gl)LEEVDEEEEEK	K(0.016),K(0.984)	3
TAF12	1.00	0.000629018	SAIFK(gl)QTEPAIPISENISTK	K(1)	3
ADH1	0.58	0.00232257	SIGGEVFIDFTK(gl)EK(x)	K(0.417),K(0.583)	3
SPP41	1.00	3.71909E-32	IPEIK(gl)NESVDLGSNITDILSSTITNILPEITATDVK	K(1)	3
SPP41	1.00	0.00829123	K(gl)IPLFNFVK	K(1)	3
TDH2	1.00	0.0059662	VVDLVEHVAK(gl)A	K(1)	3
SEF1	1.00	0.00155028	DSK(gl)VSVQTYLSR	K(1)	3
RPC53	1.00	0.00565709	FKPK(gl)AVAR	K(1)	2
NOP12	0.98	0.00038911	LLNEEAEAEDDK(gl)PTVTK(x)	K(0.985),K(0.015)	3
RPC53	1.00	4.55011E-05	SK(gl)EEREAAASK	K(1)	2
IRC20	1.00	0.00337322	K(gl)LEEADDK	K(1)	1
UME6	1.00	0.00728276	DREITDPNVK(gl)LDENESK	K(1)	3
RPS17A	1.00	0.00231406	YYPK(gl)LTLDFQTNK	K(1)	2
RSC4	1.00	0.00649697	LIAKPETVQSEVK(gl)NER	K(1)	3
NOP7	1.00	1.31632E-05	LDPTEIEEDVK(gl)VESLDASTLK	K(1)	3
RPC53	1.00	9.9783E-07	PTREPTPSVK(gl)TEPVGTGLQSYLEERER	K(1)	4
RRP15	0.97	0.00187183	LFNAILATQVK(gl)TEK(x)	K(0.97),K(0.03)	3
NET1	0.50	0.0026433	K(gl)IK(gl)SSIVEEDIVSR	K(0.5),K(0.5)	3
NET1	0.66	0.0026433	K(gl)IK(gl)SSIVEEDIVSR	K(0.339),K(0.661)	3

RPC37	1.00	0.0031902	DK**(gl)**AESEWNGVNVQTLK	K(1)	3
BUD3	1.00	0.0019115	NK**(gl)**QENINSSSNLFPEGK	K(1)	3
EMG1	1.00	9.13E-10	ISSNGPGGDK(x)	K(1)	2
SUM1	1.00	0.0131959	VNVEENK**(gl)**TEK	K(1)	2
BDF1	1.00	0.00107771	KEEGGQGTK**(gl)**QEDLDENSK	K(1)	3
BDP1	1.00	0.000245277	K**(gl)**TEVVLGTIDDLK	K(1)	3
RSC8	0.50	2.81211E-10	PFLPENVIKQEVEGGDGAEPQVK(gl)K(x)	K(0.5),K(0.5)	4
BIR1	1.00	2.10265E-09	LFDEEFSGK(gl)ELDIPIDSSTVEIK	K(1)	3
RRP9	1.00	1.08088E-06	TIDEYNNFDAGDLDK(gl)DIIASR	K(1)	3
SWC3	0.95	0.00117523	TTAESTQVDVK(gl)K(gl)EEEDVKEK	K(0.954),K(0.046)	4
SPP41	1.00	2.31495E-25	GVTTPIK(gl)IEDSDANVPPVSIAVSTIEPSQDK	K(1)	3
PRP45	1.00	0.000617658	DVSEK(gl)IILGAAK	K(1)	3
GZF3	1.00	2.48137E-09	AISNVK(gl)TETTPPHFIPFLQSSK	K(1)	4
SIR3	0.87	2.07474E-08	K(gl)IK(gl)IEPSADDDVNNGNIPSQR	K(0.866),K(0.134)	3
CDC19	1.00	1.01608E-06	IIVK(gl)IENQQGVNNFDEILK	K(1)	3
RPL5	1.00	0.000547443	VAAK(gl)IAALAGQQ	K(1)	2
GPB1	1.00	0.00768636	KENEALLK(gl)K(x)	K(1),K(1)	2
RPA43	1.00	5.72381E-05	K(gl)IVFDDEVSIENK	K(1)	3
ALY2	1.00	0.0113016	FAPLDK(gl)VTLHR	K(1)	2
PRP22	1.00	8.12604E-11	IYQIASPPVMK(gl)EEVSVLPSTK	K(1)	3
ISW1	1.00	3.86404E-05	DIISPLLLNPTK(gl)R	K(1)	3
GCD14	1.00	1.30562E-05	K(gl)RMFNNTIDSNDEK(gl)VGK(x)	K(1),K(0.02),K(0.98)	3
SPP41	1.00	7.04608E-24	RILSRPK(gl)SEDHEWPLSDSSASQNYDAHLK	K(1)	4
TUP1	1.00	1.24158E-06	APESTLK(gl)ETEPENNNTSK	K(1)	3
RPD3	1.00	0.000686863	DTK(gl)GGSQYAR	K(1)	2
YDL156W	1.00	1.28045E-09	KLHLSGVASQIK(gl)HEAGVLEK	K(1)	5
RHR2	1.00	3.42996E-24	TYDAIAK(gl)FAPDFADEEYVNKLEGEIPEK	K(1)	4
PRI1	1.00	0.00221679	NELGSVK(gl)R	K(1)	2
CBF5	1.00	2.00878E-18	EDFVIK(gl)PEAAGASTDTSEWPLLLK	K(1)	3
CIN5	0.99	2.78761E-11	KMTDTAFVPSPPVGFIK(gl)EENK(x)	K(0.994),K(0.006)	3
LIF1	1.00	1.1432E-18	ISNQSVIK(gl)MEDDDFDDFQFFGLSK	K(1)	3
BDP1	1.00	0.000245277	K**(gl)**TEVVLGTIDDLK	K(1)	3
BIR1	1.00	1.84556E-06	VIK(gl)PEFEPVPSVAR	K(1)	3
TUP1	1.00	1.37201E-06	ETTTLPSVK(gl)APESTLK	K(1)	2
NIP1	1.00	0.0108083	LEEAMVKLNK(x)	K(1)	2
RPL13B	1.00	5.71615E-09	GFTLAEVK(gl)AAGLTAAYAR	K(1)	3
SHS1	1.00	0.00303587	SIK(gl)TESSPK	K(1)	2
TOA1	1.00	3.35828E-28	IEVK(gl)PEIELTINNANITTVENIDDESEK	K(1)	3
SNF2	1.00	9.72629E-16	VAK(gl)QALDLYHFALNYENEAGRK	K(1)	4
SWR1	1.00	1.5465E-08	YDHIAK(gl)VEEPSEAFTIK	K(1)	3
SWC3	0.65	8.60728E-10	TTAESTQVDVK(gl)K(gl)EEEDVK	K(0.35),K(0.649)	3
RPC53	1.00	5.02409E-10	LADLGLEK(gl)EFQSVDGK	K(1)	3
SIZ1	1.00	9.02989E-18	TLDPK(gl)SYNIVASETTTPVTNR	K(1)	3

SWC3	0.81	1.78374E-09	SEDTQK(gl)K(gl)EDNQVVPK	K(0.806),K(0.194)	3
ABF1	1.00	2.83651E-34	VSNDSK(gl)LDFVTDDLEYHLANTHPDDTNDK	K(1)	4
CET1	0.93	2.36759E-33	KIAGNAVGSVVK(gl)K(gl)EEEANAAVDNIFEEK	K(0.934),K(0.066)	3
CET1	0.80	2.36759E-33	IAGNAVGSVVK(gl)K(gl)EEEANAAVDNIFEEK	K(0.201),K(0.799)	3
TOF2	1.00	0.000050371	LHQSQGK(gl)EALFR	K(1)	2
NTG1	1.00	3.35067E-14	LENDISVK(gl)VED	K(1)	2
PZF1	1.00	3.50961E-07	HSNEQDEEK(gl)ISNR	K(1)	3
TRI1	1.00	2.14354E-12	VLLSAPLQK(gl)FLGSEELPR	K(1)	3
UBA2	1.00	0.000194125	LLAIENLWK(gl)TR	K(1)	3
TFA1	1.00	1.11785E-11	TESNTSNDVK(gl)QESINDK	K(1)	3
YDR026C	1.00	0.000142748	YVDTEK(gl)AYLAK	K(1)	3
TYE7	0.81	3.76153E-27	LQQIIPWVASEQTAFEVGDSVK(gl)K(x)	K(0.808),K(0.192)	3
RPP1B	1.00	0.00690993	ALEGK(gl)DLK	K(1)	2
TFC3	1.00	2.69182E-13	RIK(gl)LEQHVSTAQEPK	K(1)	3
STH1	0.99	2.69935E-09	EDIEEHFK(gl)K(x)	K(0.992),K(0.008)	2
SIZ1	1.00	8.60228E-14	NFLQNALVVGK(gl)SDPYR	K(1)	2
VMA1	1.00	8.62886E-21	AIK(gl)EESQSIYIPR	K(1)	3
RVB2	1.00	2.66339E-07	RK(gl)NNTVEVEDVK	K(1)	3
POB3	0.95	3.70995E-40	KEESSNEVVPK(gl)K(gl)EDGAEGEDVQMAVEEK	K(0.949),K(0.051)	4
SGS1	1.00	6.92473E-17	ETATLQEDK(gl)DFVFQAIQK	K(1)	3
SPP41	1.00	2.78537E-48	DGDEK(gl)STLHSDAAQLTGNEPDSVNTTTGKPK	K(1)	4
GCN4	1.00	1.28118E-07	FIK(gl)TEEDPIIK	K(1)	3
SIZ1	1.00	2.74297E-45	SGLPLINNENSVPNPPNTATIPLQK(gl)SR	K(1)	3
RAP1	1.00	1.56505E-38	DSIRPK(gl)TEIISTNTNGATEDSTSEK	K(1)	3
MLP2	1.00	5.5127E-28	RVK(gl)EEYDIWQSRDQGNDSLNDDLNK	K(1)	4
CDC3	1.00	9.30413E-08	LGIK(gl)QDNSVFK	K(1)	3
RPS1A	1.00	4.24111E-09	VTGFK(gl)DEVLETV	K(1)	2
LEU2	1.00	2.85531E-13	TVEETIK(gl)NEFPTLK(x)	K(0.999),K(0.001)	3
BDP1	1.00	1.35101E-29	ARQEFKPLHSLTK(gl)EEQEEEEEK	K(1)	4
RAD16	1.00	1.61213E-11	NDNDEIIEIK(gl)EER	K(1)	3
PGI1	1.00	9.03644E-07	TLSVK(gl)QEFQK	K(1)	2
RP	1.00	5.54633E-28	TLSDYNIQK(gl)ESTLHLVLR	K(1)	3
PRP45	1.00	4.33206E-25	K(gl)QTSTVAR	K(1)	2
PRP45	0.90	4.33206E-25	YHNGTPQTGAIVK(gl)PK(gl)K(x)	K(0.02),K(0.898),K(0.082)	3
TOP2	0.93	2.52243E-36	TPSVSETKTEEEENAPSSTSSSSIFDIK(gl)K(gl) EDK(x)	K(0.929),K(0.055),K(0.017)	4
TOP2	0.50	6.11243E-35	TEEEENAPSSTSSSSIFDIK(gl)K(x)	K(0.5),K(0.5)	2
RPL4A	1.00	9.20286E-14	LNPYAK(gl)VFAAEK	K(1)	2
TUP1	0.96	5.15374E-17	LWNLQNANNK(gl)SDSK(x)	K(0.965),K(0.035)	3
UBC9	0.80	1.74618E-53	EGTNWAGGVYPITVEYPNEYPSK(gl)PPK(gl)VK(x)	K(0.036),K(0.167),K(0.797)	4
TOP1	0.98	1.22231E-09	KIK(gl)K(gl)EDGDVK	K(0.978),K(0.022)	2
RPL25	1.00	6.57184E-28	LDSYK(gl)VIEQPITSETAMK	K(1)	3

SWR1	1.00	8.10672E-15	AGGEQDLADLK(gl)FR	K(1)	3
PGK1	1.00	1.25571E-09	VK(gl)ASKEDVQK	K(1)	2
RPL34B	1.00	1.25491E-11	AFLIEEQK(gl)IVK	K(1)	3
EBP2	0.98	1.30299E-28	SQELK(gl)K(gl)EEPTIVTASNLK	K(0.979),K(0.021)	3
TFG1	1.00	0.00194738	K(gl)IDEDGER	K(1)	2
STP1	1.00	0.000921968	IK(gl)SEVNAK	K(1)	2
VPS72	1.00	2.89595E-43	VNSDELK(gl)PTALPDVTLDAIANK	K(1)	3
RAD59	0.99	4.96659E-55	NEANTNYNLLSATNSK(gl)PTFIK(gl)LEDAK(x)	K(0.007),K(0.989),K(0.004)	4
TYE7	1.00	5.29064E-19	TNLDAK(gl)ETK	K(1)	2
NTG1	1.00	8.89716E-33	IK(gl)QEEVVPQPVDIDWVK	K(1)	3
SMC5	1.00	4.61581E-16	LDDIVSK(gl)ISAR	K(1)	2
POL30	0.95	1.13232E-46	IVRDLSQLSDSINIMITK(gl)ETIK(x)	K(0.047),K(0.953)	4
YSH1	1.00	6.34494E-47	DEYASNK(gl)EETITGVVTIGK	K(1)	3
VMA2	0.50	8.90406E-37	AIVQVFEGTSGIDVK(gl)K(x)	K(0.5),K(0.5)	3
PRP45	1.00	8.12413E-57	LDEAVNVK(gl)SEGASGSHGPIQFTK	K(1)	3
GLO2	1.00	1.4606E-27	FTLK(gl)DEVEFNPFMR	K(1)	3
NET1	1.00	5.64303E-37	VADLK(gl)SANIGGEDLNK	K(1)	3
NGG1	0.97	5.13196E-37	LGPLYTDVWFK(gl)DENDK(x)	K(0.971),K(0.029)	3
CBF5	0.99	2.76039E-20	VNENTPEQWK(gl)K(x)	K(0.99),K(0.01)	2
BIR1	1.00	1.04704E-79	ILEDVSVK(gl)NETPNNEMLLFETGTPIASQENK	K(1)	3
SPT15	1.00	2.86803E-43	DGTK(gl)PATTFQSEEDIK	K(1)	2
TYE7	1.00	1.86653E-89	SSETTLIK(gl)PESEFDNWLSDENDGASHINVNK	K(1)	3
TFG1	1.00	2.19244E-81	AVDSSNNASNTVPSPIK(gl)QEEGLNSTVAER	K(1)	3
FBA1	1.00	6.14851E-51	DYIMSPVGNPEGPEK(gl)PNK(x)	K(0.998),K(0.002)	3
DEP1	0.99	2.99449E-31	SQELEEAIDSK(gl)EK(x)	K(0.995),K(0.005)	3
SPP41	0.98	2.3859E-31	NYQYEDENVK(gl)YLK(x)	K(0.976),K(0.024)	3
SIZ1	1.00	3.61342E-45	STNTDILTEK(gl)GSSAPSR	K(1)	3
TOP1	1.00	1.23198E-31	AK(gl)EEEEEYKWWEK	K(1)	3
RSC8	1.00	1.90805E-67	PFLPENVIK(gl)QEVEGGDGAEPQVKK	K(1)	4
BUD4	1.00	1.3352E-84	QENNEINIK(gl)AEEEIEPMTQQETDGLK	K(1)	3
ISW1	1.00	5.17381E-59	AK(gl)IEDTSNVGTEQLVAEK	K(1)	3
RPS1B	1.00	4.0053E-36	VSGFK(gl)DEVLETV	K(1)	2
YSH1	1.00	1.76976E-58	IEPIK(gl)EENEDNLDSQAEK	K(1)	2
SRS2	1.00	9.85497E-25	VK(gl)VEEVIDLK	K(1)	2
RPS21B	1.00	1.89114E-66	ADDHASVQINVAK(gl)VDEEGR	K(1)	3
RPS3	1.00	7.95569E-78	ALPDAVTIIEPK(gl)EEEPILAPSVK	K(1)	3
REP2	1.00	1.21025E-41	GAYK(gl)LQNTITEGPK	K(1)	2
NOP56	1.00	2.18809E-59	PTLK(gl)NELAIQEAMELYNK	K(1)	3
IES4	1.00	1.82232E-53	GSEFTASDVK(gl)GSDDK	K(1)	2
TUP1	1.00	1.59656E-29	LQNQK(gl)DYDFK	K(1)	3
MRP8	1.00	1.28319E-18	EFK(gl)DIPDLK	K(1)	2
IES4	1.00	6.40435E-50	EPADEDPEVK(gl)QLEK	K(1)	2

CDC3	1.00	4.76908E-43	LQK(gl)SETELFAR	K(1)	3
RPC82	1.00	5.97625E-68	LK(gl)TEDGFVIPALPAAVSK	K(1)	3
ISW1	1.00	4.15076E-77	ADSK(gl)DALLSMIQHGAADVFK	K(1)	3
RPC11	1.00	5.33987E-69	K(gl)EVDDVLGGGWDNVDQTK	K(1)	3
SKO1	1.00	4.62602E-91	DTNVVK(gl)SENAGYPSVNSRPIILDK	K(1)	3
TRI1	1.00	4.43993E-58	EIK(gl)LENESLPNLSG	K(1)	2
YLR455W	1.00	7.39377E-59	NSISIK(gl)EDPEDNQK	K(1)	3
VPS72	1.00	1.0636E-78	SDIK(gl)RDETTNEDSDDQVRFK	K(1)	4
MOT1	1.00	1.61666E-71	TDDIK(gl)QETSMLNASDK	K(1)	3
CDC3	1.00	5.194E-110	FEAAESDVK(gl)VEPGLGMGITSSQSEK	K(1)	3
SIR4	1.00	7.7439E-79	APFIK(gl)SESKPFSSDALSK	K(1)	3
POL30	1.00	5.7235E-119	LMDIDADFLK(gl)IEELQYDSTLSLPSSEFSK	K(1)	4
TRI1	1.00	7.25646E-72	HLFNPDEIVK(gl)HEEEQK	K(1)	3
NFI1	1.00	2.31361E-72	NENQGTVK(gl)QEQDYDSRNAFDTNLR	K(1)	4
NCB2	1.00	1.18544E-82	SRLHHNSVSDPVK(gl)SEDSS	K(1)	3
TUP1	1.00	1.73266E-51	DAYEEEIK(gl)HLK	K(1)	2
SDC1	1.00	3.7948E-124	SVTNQNVK(gl)IEESSSTNSVIEESSEPK	K(1)	3
RNR2	1.00	3.1895E-75	STK(gl)QEAGAFTFNEDF	K(1)	2
MLP1	0.99	5.59797E-67	K(gl)IK(gl)TEDEEEK	K(0.013),K(0.987)	3
CET1	0.98	3.47793E-90	RALSLDDLVNHDENEK(gl)VK(x)	K(0.981),K(0.019)	3
RPS0B	1.00	7.7098E-46	TWEK(gl)LVLAAR	K(1)	2
RP	1.00	7.43999E-59	LIFAGK(gl)QLEDGRTLSDYNIQK	K(1)	3
ZEO1	1.00	3.80596E-67	NEATPEAEQVK(gl)K(x)	K(0.995),K(0.005)	2
TDH2	1.00	1.2946E-110	TASGNIIPSSTGAAK(gl)AVGK(x)	K(0.999),K(0.001)	3
RAD52	1.00	1.39087E-95	K**(gl)**PVFGNHSEDIQTKLDK	K(1)	3
SOD1	1.00	1.66982E-60	K**(gl)**THGAPTDEVR	K(1)	2
TFG1	1.00	7.5828E-68	K**(gl)**DDPEYAEEREK	K(1)	2
RPS8A	1.00	5.38192E-55	NVK**(gl)**EEETVAK	K(1)	2
SHS1	1.00	3.8171E-61	QLGREIK**(gl)**QENENLIR	K(1)	3
RPC53	1.00	2.663E-155	MAK**(gl)**YLNNTHVISSGPLAAGNFVSEK	K(1)	3
NTG1	1.00	6.7066E-116	RPLVK**(gl)**TETGPESELLPEK	K(1)	4
RVB1	1.00	3.67647E-78	K**(gl)**EIVVNDVNEAK	K(1)	2
RPC53	1.00	7.89719E-56	DTK**(gl)**DALSTR	K(1)	2
BDP1	1.00	7.24043E-88	KGSGGIMTNDLK**(gl)**VYR	K(1)	3
BDP1	1.00	5.51277E-88	DK(gl)LLNADIPESDRK	K(1)	3
RPS10B	1.00	1.64306E-91	HEEIDTK(gl)NLYVIK	K(1)	3
RPC53	1.00	1.99736E-98	VK(gl)LEEESK	K(1)	2
VHR1	1.00	1.25565E-75	NLFNIINK(gl)NK(x)	K(0.998),K(0.002)	2
NET1	1.00	2.6415E-139	SDLFK(gl)MIEGDDTDLPQWFK	K(1)	3
RPC53	1.00	6.0422E-139	LPAFERPAVK(gl)EEK	K(1)	2
ZPR1	1.00	6.4831E-99	EQNEDLGLSDIK(gl)VE	K(1)	2

TFG1	1.00	6.3772E-120	GSLVK(gl)K(gl)DDPEYAEEREK	K(1),K(1)	3
RPP2A	1.00	1.1752E-164	MK(gl)YLAAYLLLNAAGNTPDATK	K(1)	3
NTG1	1.00	2.1829E-131	RELNVEAEINVK(gl)HEEK	K(1)	3
SUM1	1.00	1.2964E-120	SDASNRIK(gl)NEIPINSLLPSSK	K(1)	4
HAP1	1.00	8.27484E-88	VK(gl)QESSDELKKDDFMK	K(1)	4
TAF14	1.00	4.0511E-134	TGSASTVK(gl)GSVDLEK	K(1)	2
BDP1	0.99	2.00501E-90	LNDANLNK(gl)K(x)	K(0.991),K(0.009)	2
HTB1	1.00	8.8792E-108	AVTK(gl)YSSSTQA	K(1)	2
SPP41	0.89	1.4956E-204	STLHSDAAQLTGNEPDSVNTTTGK(gl)PK(x)	K(0.885),K(0.115)	3
HMO1	1.00	1.04648E-91	TTDPSVK(gl)LK	K(1)	3
RPL8A	1.00	1.0916E-129	NFGIGQAVQPK(gl)R	K(1)	2
SPT15	1.00	1.6355E-165	PATTFQSEEDIK(gl)R	K(1)	3
YDL156W	1.00	3.2125E-160	LSDLIK(gl)DEDESALLEK	K(1)	3
RET1	1.00	1.0172E-215	HVK(gl)DEAFDDLLKPVYK	K(1)	3
RPL18A	1.00	7.7552E-115	ALK(gl)QEGAANK	K(1)	2
ABF1	1.00	3.2284E-161	QQGVTIK(gl)NDTEDDSINK	K(1)	2
TFA2	0.99	1.4476E-104	NPVLVDIK(gl)K(x)	K(0.995),K(0.005)	2
CET1	1.00	3.7133E-177	RDLEVLNEISASSK(gl)PSK	K(1)	3
SUM1	1.00	9.8824E-242	IITIK(gl)SSSENSGNNTTNNNNTDNVIK	K(1)	3
PAA1	1.00	2.6654E-134	ELIK(gl)EEYDN	K(1)	2
GCN5	1.00	9.7815E-219	VK(gl)LENNVEEIQPEQAETNK	K(1)	3
RAD52	1.00	2.0705E-153	NLVK(gl)IENTVSR	K(1)	2
TUP1	1.00	1.1962E-195	DYDFK(gl)MNQQLAEMQQIR	K(1)	3
NET1	1.00	1.4513E-187	AK(gl)NESAQIDR	K(1)	2
RPC37	1.00	4.08E-302	SEEVK(gl)AEDDTGEEEEDDPVIEEFPLK	K(1)	3
UBC9	1.00	8.8811E-181	VLLQAK**(gl)**QYSK	K(1)	2
UBA2	1.00	4.2688E-228	RIK**(gl)**QETNELYELQK	K(1)	3
SPP41	1.00	3.9106E-291	RPQIK**(gl)**PEVSVINLVQNLVNTK	K(1)	3
BOP3	1.00	0	IGASAVAALNDNISIK**(gl)**EEDVAR	K(1)	3
CDC48	1.00	0	EVK**(gl)**VEGEDVEMTDEGAK	K(1)	3
BUD3	1.00	7.43E-262	FFEIEEELK**(gl)**EELK	K(1)	3
TFG1	1.00	1.2275E-303	VK**(gl)**DEDPNEYNEFPLR	K(1)	3
RPC53	1.00	0	RGFIK**(gl)**SEGSGSSLVQK	K(1)	3
BDP1	1.00	0	NTAK**(gl)**EEDQTAQR	K(1)	2
CDC3	1.00	0	SLKEEQVSIK**(gl)**QDPEQEER	K(1)	3
HPC2	0.50	0	MQTQTDTNAEVLNTDNSIK(gl)K(x)	K(0.5),K(0.5)	3
RSC58	1.00	0	VK(gl)QEELLNTNEEGINR	K(1)	3
DEP1	1.00	0	LSSLVK(gl)QETLTESLK	K(1)	2
POL30	1.00	0	DLSQLSDSINIMITK(gl)ETIK	K(1)	2
YDL156W	1.00	0	IFLFTDDSGTIK(gl)QEE	K(1)	3
CRZ1	1.00	0	IESGIVNIK(gl)NELDDTSK	K(1)	2
REB1	1.00	0	ELVDYFSSNISMK(gl)TEN	K(1)	2
